# Bioenergetic Failure Drives Functional Exhaustion of Monocytes in Acute-on-Chronic Liver Failure

**DOI:** 10.3389/fimmu.2022.856587

**Published:** 2022-06-03

**Authors:** Deepanshu Maheshwari, Dhananjay Kumar, Rakesh Kumar Jagdish, Nidhi Nautiyal, Ashinikumar Hidam, Rekha Kumari, Rashi Sehgal, Nirupama Trehanpati, Sukriti Baweja, Guresh Kumar, Swati Sinha, Meenu Bajpai, Viniyendra Pamecha, Chhagan Bihari, Rakhi Maiwall, Shiv Kumar Sarin, Anupam Kumar

**Affiliations:** ^1^ Department of Molecular and Cellular Medicine, Institute of Liver and Biliary Sciences, New Delhi, India; ^2^ Department of Hepatology, Institute of Liver and Biliary Sciences, New Delhi, India; ^3^ Department of Obstetrics and Gynaecology, Sitaram Bhartia Institute of Science and Research, New Delhi, India; ^4^ Department of Transfusion Medicine, Institute of Liver and Biliary Sciences, New Delhi, India; ^5^ Department of Hepato-Pancreato-Biliary (HPB) Surgery and Liver Transplant, Institute of Liver and Biliary Sciences, New Delhi, India; ^6^ Department of Pathology, Institute of Liver and Biliary Sciences, New Delhi, India

**Keywords:** bioenergetics, ucMSC therapy, regeneration, acute-on-chronic liver failure (ACLF), monocyte

## Abstract

**Objective:**

The monocyte–macrophage system is central to the host’s innate immune defense and in resolving injury. It is reported to be dysfunctional in acute-on-chronic liver failure (ACLF). The disease-associated alterations in ACLF monocytes are not fully understood. We investigated the mechanism of monocytes’ functional exhaustion and the role of umbilical cord mesenchymal stem cells (ucMSCs) in re-energizing monocytes in ACLF.

**Design:**

Monocytes were isolated from the peripheral blood of ACLF patients (*n* = 34) and matched healthy controls (*n* = 7) and patients with compensated cirrhosis (*n* = 7); phagocytic function, oxidative burst, and bioenergetics were analyzed. In the ACLF mouse model, ucMSCs were infused intravenously, and animals were sacrificed at 24 h and day 11 to assess changes in monocyte function, liver injury, and regeneration.

**Results:**

Patients with ACLF (alcohol 64%) compared with healthy controls and those with compensated cirrhosis had an increased number of peripheral blood monocytes (*p* < 0.0001) which displayed significant defects in phagocytic (*p* < 0.0001) and oxidative burst capacity (*p* < 0.0001). ACLF patients also showed a significant increase in the number of liver macrophages as compared with healthy controls (*p* < 0.001). Bioenergetic analysis showed markedly reduced oxidative phosphorylation (*p* < 0.0001) and glycolysis (*p* < 0.001) in ACLF monocytes. Patients with monocytes having maximum mitochondrial respiration of <37.9 pmol/min [AUC = 0.822, hazard ratio (HR) = 4.5] and baseline glycolysis of ≤42.7 mpH/min (AUC = 0.901, HR = 9.1) showed increased 28-day mortality (*p* < 0.001). Co-culturing ACLF monocytes with ucMSC showed improved mitochondrial respiration (*p* < 0.01) and phagocytosis (*p* < 0.0001). Furthermore, ucMSC therapy increased monocyte energy (*p* < 0.01) and phagocytosis (*p* < 0.001), reduced hepatic injury, and enhanced hepatocyte regeneration in ACLF animals.

**Conclusion:**

Bioenergetic failure drives the functional exhaustion of monocytes in ACLF. ucMSCs resuscitate monocyte energy and prevent its exhaustion. Restoring monocyte function can ameliorate hepatic injury and promote liver regeneration in the animal model of ACLF.

## Introduction

Acute-on-chronic liver failure (ACLF) is a life-threatening condition caused by acute hepatic injury in patients with underlying chronic liver disease resulting in liver failure (1–6). Unresolved injury, poor infection control, and liver regeneration result in persistent systemic inflammation and cytokine storm, which subsequently lead to systemic inflammatory response syndrome (SIRS) resulting in multiple organ failure, septic shock, and death in ACLF (1, 4, 5, 7–9). Nearly 74% of ACLF patients initially diagnosed without SIRS or sepsis develop SIRS by day 7 which increases the onset of secondary organ failure and sepsis with high short-term mortality (9). About 25%–37% of these patients show the presence of infection/sepsis at the time of presentation and nearly 46% of the remaining patients develop infection/sepsis by day 28, which further increases secondary organ failure and lowers the 90-day probability of survival (9–12). The precise mechanism of poor resolution of infection and ongoing hepatic injury that accounts for SIRS and the subsequent onset of sepsis and secondary organ failure in ACLF is not clear.

Liver macrophages [Kupffer cells (KCs)] along with bone marrow (BM) monocytes play an important role in the host’s innate immune defense against pathogens and injury (13–16). Although the number of KCs increases in ACLF compared to healthy (8), they fail to clear the infection and resolve the injury. Previous studies have shown that monocytes in ACLF show a suppressive phenotype with reduced human leucocyte antigen-DR (HLA-DR) expression, antigen presentation, and impaired pro-inflammatory cytokine production in response to bacterial components (17–21). These cells also show significantly low expression of toll-like receptors 2/4 (TLR-2/TLR-4) and defects in their phagocytic and oxidative burst functions (22) necessary for the identification, engulfment, and killing of bacteria, respectively. The underlying cause for these defects is still not clear.

Functional heterogeneity of monocytes/macrophages is regulated at the transcriptional and metabolic levels (23). Monocytes/macrophages adopt a distinct metabolic pathway upon encountering pathogen/tissue damage and use a variety of carbon sources to fuel their diverse functions (24). In response to infection or necrotic stimuli, these cells mainly rely on glycolysis for ATP requirement (25) and execute their pro-inflammatory and bacterial killing function by remodeling the TCA cycle and electron transport chain (26–28). On the other hand, anti-inflammatory monocytes maintain basal levels of glycolysis for sustaining oxidative phosphorylation (OXPHOS) but prefer fatty acid oxidation for producing ATP and anti-inflammatory cytokines such as IL-10 (25, 29–31).

Monocyte functions such as phagocytosis, efferocytosis, and oxidative burst are highly energy-demanding processes. Infections are associated with broad defects in energy metabolism in a dose-dependent manner leading to immune paralysis as seen in sepsis (32, 33). ACLF patients have intestinal barrier failure which may increase the exposure of monocytes to bacteria and bacterial products (34–36). In ACLF, it is unclear whether there is metabolic dysfunction or metabolic exhaustion of monocytes, responsible for the poor response to infection. In this study, we aimed to analyze the changes in the energy metabolism of monocytes during ACLF progression and their impact on monocyte functions. We also investigated whether restoring ACLF monocyte bioenergetics by using mesenchymal stem cells (MSCs) could resuscitate their phagocytic function. These results may provide new insights into the pathophysiology of innate immune dysfunction and the therapeutic use of MSCs for the management of ACLF.

## Materials and Methods

### Patients

We recruited ACLF patients (*n* = 34) from the Institute of Liver and Biliary Sciences, India, between July 2017 and January 2021. Patients were characterized according to the Asian Pacific Association for the Study of the Liver (APASL)-ACLF criteria (2). Patients were excluded based on the following criteria: were <18 years of age; had autoimmune hepatitis, septic shock, extrahepatic malignancies, or prior liver transplant; were pregnant; and did not provide consent. Patients with ACLF were stratified into ACLF-systemic inflammatory response syndrome (ACLF-SIRS) (SIRS is defined as the presence of any of the two criteria from the following: temperature >104.4 or <96.8°F, heart rate >90/min, respiratory rate >20/min, TLC >12), ACLF-sepsis (infection with or without SIRS), and ACLF (having no SIRS and infection). Baseline blood was collected at the time of admission before initiating therapy. In consenting patients, blood was also collected at 24 and 72 h to study the cellular kinetics. Blood and urine cultures were obtained, ascitic fluid was collected for analysis, and a chest X-ray was performed to confirm infections and pulmonary infiltrates as per standard care. The blood from age- and gender-matched healthy volunteers (*n* = 7) and patients with compensated cirrhosis (*n* = 7) was taken as the control for the analysis of monocyte energy and function.

Explant liver tissues from ACLF patients (*n* = 8) who had undergone live donor liver transplant (LDLT) and liver tissues of healthy controls [*n* = 5; cadaveric liver unfit for transplant = 1; discarded donor liver tissue after trimming during adult to pediatric (>2 months) LDLT = 4] were collected to analyze the distribution and function of liver macrophages.

Written informed consent was obtained from all patients or their designated family members. The study protocol conformed to the ethical guidelines of the 1975 Declaration of Helsinki as reflected in the *a-priori* approval by the appropriate institutional review committee. No donor organs were obtained from executed prisoners or other institutionalized persons.

### Monocyte Isolation and Culture

Blood was collected from healthy donors and ACLF patients in EDTA-coated vacutainers (BD Biosciences, New Jersey, USA). Plasma was separated and collected for further analysis. CD14^+^ monocytes were isolated using a positive selection procedure according to the manufacturer’s specification (Miltenyi CD14 MicroBeads, human, Miltenyi Biotec, Bergisch Gladbach, Germany). For bioenergetic analysis, freshly isolated monocytes were used. Monocytes were cultured in RPMI medium (Gibco, USA) containing antibiotics (1% penicillin and streptomycin) and further supplemented with either plasma from healthy donors or plasma from patients with ACLF at a final concentration of 10% by volume with or without LPS (Sigma Aldrich, Missouri, USA) (100 ng/ml) to study the effect of ACLF plasma and endotoxins on the energy metabolism of healthy monocytes (22). Patient plasma used in these experiments represents a pool of six healthy donors or ACLF (without SIRS or sepsis). Plasma was heat-inactivated (56 C for 30 min) and passed through a 0.22-μM filter prior to use. Following a 6-h incubation time (37 C, 5% CO_2_), the cells were harvested and used for bioenergetic analysis. To study the effect of glycolysis and OXPHOS on monocyte phagocytic and oxidative burst function, cells were pre-incubated with RPMI medium containing 1.0 μM oligomycin, 0.75 μM FCCP, or 0.5 μM rotenone + antimycin A for the inhibition of OXPHOS or 50 mM 2-deoxy-2-glucose (2-DG) for the inhibition of glycolysis for 30 min (37 C, 5% CO_2_), and then monocytes were analyzed for phagocytic capacity (Cayman Chemicals, Michigan, USA) and ROS production (Sigma Aldrich, Missouri, USA). Pre- and post-treatment cells were stained with annexin V to analyze the effect of OXPHOS and glycolysis inhibitors on cell death using a flow cytometer.

### Cellular Bioenergetic Studies

Oxygen consumption rate (OCR) and extracellular acidification rate (ECAR) were measured using Seahorse XFe24 Extracellular Flux Analyzer (Agilent Technologies, California, USA) as readout for OXPHOS and glycolysis, respectively. An equal number (3 × 10^5^/well) of viable CD14^+^ monocytes (as assessed by trypan blue dye exclusion assay) were seeded on CellTak (Corning)-coated plates in XF-Base Media (Agilent Technologies) containing 2.5 mM glucose, 1 mM sodium pyruvate, and 2 mM glutamine. OCR and ECAR were measured sequentially at the basal level and following the addition of 1.0 μM oligomycin, 0.75 μM FCCP (fluorocarbonyl cyanide phenylhydrazone), and 0.5 μM rotenone + antimycin to analyze changes in glycolysis and various mitochondrial respiratory parameters. The plate was read three times each at baseline and after the addition of drugs. Data were analyzed using Wave 2.6.1 and GraphPad Prism.

### Monocyte–Mesenchymal Stem Cell Co-Culture

Prior to monocyte co-culture, MSCs were seeded in Transwell inserts at a ratio of 1:5 (MSCs to monocytes) and incubated in complete alpha-MEM (containing 10% FBS, 1% GlutaMAX, 1% non-essential amino acids, and 1% PSA) for 24 h. MSCs in Transwell were co-cultured with monocytes in TexMACS™ Medium (Miltenyi Biotec, California, USA) (without MCSF) for 24 h. After 24 h, monocytes were harvested and analyzed for mitochondrial bioenergetics using Seahorse XFe24 Extracellular Flux Analyzer (Agilent Technologies). Cells were also analyzed for mitochondrial biomass and mitochondrial membrane potential using MitoTracker dyes. To study the mitochondria transfer from MSC to monocyte, MSCs were pre-incubated with 300 nm MitoTracker Red CMXRos (Life Technologies, California, USA) for 2 h and washed three times with PBS to remove any unbound dye. MSCs were then co-cultured with monocytes for 24 h. The cells were then assayed for fluorescence by BD FACSAria and by confocal microscopy.

### ACLF Murine Model and MSC Administration

Six- to 8-week-old male C57Bl6 mice (Liveon Biolabs Pvt. Ltd., Karnataka, India) were used to develop the animal model of ACLF as described previously (37). All the animals were housed in the institutional animal facility in a clean, temperature-controlled environment with a 12-h light and dark cycle and were provided with free access to regular laboratory chow diet and water. All animals received humane care according to the criteria outlined in the “Guide for the Care and Use of Laboratory Animals,” and all experimental procedures were approved by the Institutional Animal Ethics Committee (IAEC) under project approval (IAEC/ILB/17/03). To develop the ACLF animal model, 8–10-week-old male C57BL6/J mice (*n* = 24) were first subjected to carbon tetrachloride (CCl_4_)-induced chronic liver injury for 10 weeks. Following 10 weeks of chronic liver injury, a single dose of acetaminophen (350 mg/kg bw) and LPS (100 μg/kg bw) was given to induce ACLF. Sixteen hours post-ACLF injury, animals were divided into two groups. Group 1 received ucMSCs (1 × 10^6^/kg wt), while group 2 received 200 μl PBS as a control. Mice were sacrificed by an overdose of ketamine post-24 h and day 11 of cell therapy, blood was collected through retro-orbital bleeding, and BM and liver were harvested for cell isolation and histology.

### Statistical Analysis

Data were analyzed using SPSS version 22 (IBM Corp. Ltd., USA). Categorical data were compared using chi-square test and continuous data were compared by using one-way ANOVA/Kruskal–Wallis test, wherever applicable followed by *post-hoc* comparison by the Bonferroni method. Log transformation was applied wherever necessary. Univariate and multivariate analyses were carried out using multinomial logistic regression and expressed as odds ratio (OR, 95 CL). Diagnostic tests were applied to find out the area under the curve (AUROC) and Kaplan–Meier was used to find out the survival probabilities. Statistical analysis and graphical generation of data were done with GraphPad Prism software (San Diego, CA, USA). The exact values of *n* (the number of one sample or an individual animal) and statistical significance are depicted in the figures. Error bars represent standard deviation of the mean (SD). Significant difference in means is indicated as follows: *p < 0.05, **p < 0.01, ***p < 0.001, and ****p < 0.0001.

Detailed methodology for the development of the ACLF animal model; isolation, culture, and characterization of umbilical cord mesenchymal stem cells; isolation of monocytes from peripheral blood and their phenotypic characterization; monocyte–MSC co-culture; phagocytic and oxidative burst assay; mitochondrial staining; estimation of plasma cytokines and endotoxin; and histopathology, immunohistochemistry, and TUNEL assay are provided in the Online **Supplementary Methods**.

### Study Approval

All experiments were conducted in accordance with the National Ethical Guidelines for Biomedical and Health Research and the Committee for the Purpose of Control and Supervision of Experiments on Animals (CPCSEA) Govt. of India and were approved by the Ethics Committee of the Institute of Liver and Biliary Sciences, New Delhi, India (IEC/2017/50/NA02; IAEC/ILB/17/03; 9/ILBS/IC-SCR/2017).

## Results

### Patient Demographics

Of the 34 ACLF patients, 13 had SIRS (ACLF-SIRS), 13 had sepsis (ACLF-sepsis), and 8 had neither sepsis nor SIRS (ACLF). The clinical characteristics with circulating biochemical and immunological parameters of the ACLF cohort are summarized in **Supplementary Table S1**. Eight of the 34 ACLF patients underwent LDLT. While blood was obtained from all 34 patients, liver tissue could be obtained from those who underwent LDLT. Of the seven cirrhosis patients (median age 64 years), five patients had cirrhosis secondary to alcoholic liver disease (ALD; 71.4%), one secondary to non-alcoholic fatty liver disease (NAFLD; 14.3%), and one cryptogenic (14.3%) with a median model for end-stage liver disease prognostic score of 9.1 [interquartile range (IQR) 7.1–10.2].

### Defective Phagocytic and Oxidative Burst Functions of Peripheral Blood Monocyte and Liver Macrophages in ACLF

**Figure 1:**
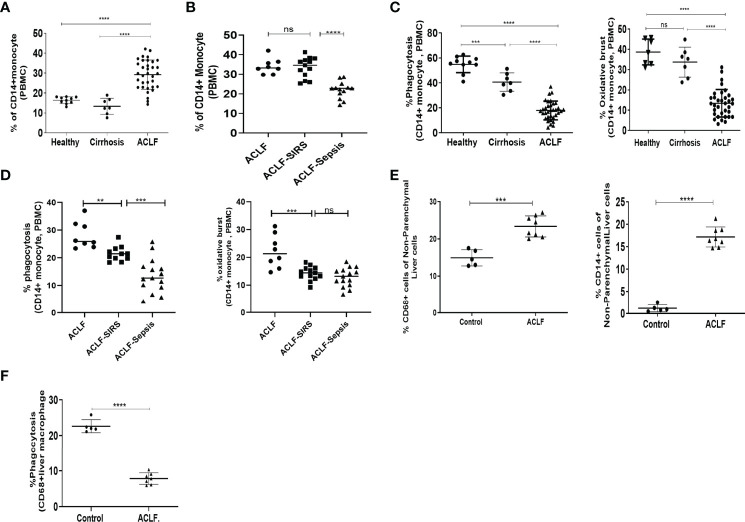
Distribution and function of peripheral blood monocytes and liver macrophages in healthy controls and acute-on-chronic liver failure (ACLF) patients. **(A)** Dot plot showing changes in the percentage of peripheral blood CD14^+^ monocytes in ACLF compared with healthy controls and those with cirrhosis. **(B)** Dot plot showing changes in the percentage of peripheral blood CD14^+^ monocytes between subgroups of ACLF (ACLF, ACLF-SIRS, and ACLF-sepsis). **(C)** Dot plot showing the percentage changes in phagocytosis (left) and oxidative burst (right) of peripheral blood CD14^+^ monocytes in ACLF compared with healthy controls and those with cirrhosis. **(D)** Dot plot showing the percentage changes in phagocytosis (left) and oxidative burst (right) of peripheral blood CD14^+^ monocytes between subgroups of ACLF (ACLF, ACLF-SIRS, and ACLF-sepsis). **(E)** Dot plot showing the percentage changes in the distribution of CD68^+^ macrophage (left) and CD14^+^ monocyte (right) in ACLF compared with healthy controls. **(F)** Dot plot showing the percentage changes in phagocytosis of CD68^+^ liver macrophages in ACLF compared with healthy controls (*p < 0.05; **p < 0.01; ***p < 0.001; ****p < 0.0001). n.s. stands for non-significant.

To identify the underlying cause of monocyte dysfunction, we first evaluated the changes in peripheral blood monocyte distribution and their phagocytic and oxidative burst function with SIRS and sepsis. Flow cytometric analysis of monocyte distribution showed a significant increase in monocytes in ACLF compared with healthy monocytes (*p* < 0.0001) and compensated cirrhosis (*p* < 0.0001) (**Figure 1A**), which was mainly contributed by intermediate monocytes (**Figures S1A, B**). Within the ACLF cohort, the number of monocytes was comparable between ACLF and ACLF-SIRS but was significantly (*p* < 0.0001) decreased in ACLF-sepsis (**Figure 1B**).

Next, we assessed the phagocytic and oxidative burst capacity of these cells on exposure to *Escherichia coli*. ACLF monocytes featured a significantly impaired phagocytic (*p* < 0.0001) and oxidative burst (*p* < 0.0001) capacity compared with healthy monocytes (*p* < 0.0001) and compensated cirrhosis (*p* < 0.0001) (**Figure 1C**). In comparison to healthy monocytes, there was a significant decrease in monocyte phagocytosis in cirrhosis which further decreased in ACLF (**Figure 1C**). Within the ACLF cohort, in comparison to ACLF, the monocytes from ACLF-SIRS showed a significant decrease in their phagocytosis (*p* < 0.01) and oxidative burst (*p* < 0.001). Between ACLF-SIRS and ACLF-sepsis, while phagocytosis further decreased (*p* < 0.001) in ACLF-sepsis, oxidative burst was comparable (**Figure 1D**), suggesting the loss of monocyte phagocytic and oxidative burst function with the onset of SIRS and sepsis.

To further understand monocyte–macrophage dysfunction in ACLF, we compared the distribution and phagocytic function of liver macrophages/KCs in ACLF with healthy monocytes (*n* = 5). Flow cytometric analysis of liver cells showed a significant (*p* < 0.001) increase in the number of CD68^+^ KCs and CD14^+^ monocytes in ACLF (**Figures 1E**, **S1C**). Out of the total CD68^+^ KCs, while the number of yolk-sac KCs (YC-KC; CD68^+^Cd14^−^CD16^−^) significantly (*p* < 0.001) decreased, the number of BM-monocyte-derived KCs (BM-KC; CD68^+^CD14^+^/CD16^+^) was significantly (*p* < 0.01) increased in ACLF (**Figure S1D**). Furthermore, CD68^+^ KCs also showed significant (*p* < 0.0001) defects in phagocytosis (**Figure 1F**), suggesting that, though the number of macrophages increases in ACLF liver, these cells were defective in their phagocytic function.

### Broad Defect in Energy Metabolism Drives the Loss of Phagocytic and Oxidative Burst Function in ACLF Monocytes

**Figure 2:**
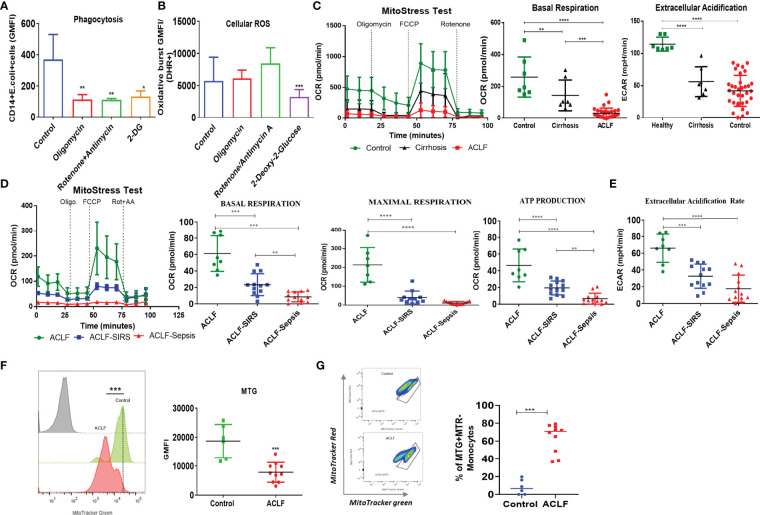
Energy metabolism of healthy and ACLF monocytes. **(A, B)** Bar graph showing changes in median fluorescence intensity of **(A)** phagocytosis and **(B)** cellular ROS of healthy CD14^+^ monocytes in the presence of inhibitors of mitochondria and glycolysis. **(C)** Real-time changes in oxygen consumption rate (OCR) with subsequent treatment with oligomycin (Oligo.) FCCP and rotenone and antimycin A (Rot. + AA.) in ACLF, cirrhosis, and healthy monocytes (left). Dot plot showing changes in basal mitochondrial respiration (middle) and glycolysis (right) in ACLF monocytes compared with healthy controls and those with cirrhosis. **(D)** Real-time changes in OCR and dot plot showing changes in the given mitochondrial respiratory parameter of monocytes in the ACLF subgroups (ACLF, ACLF-SIRS, and ACLF-sepsis). **(E)** Dot plot showing changes in glycolysis (extracellular acidification rate) of monocytes in the ACLF subgroups (ACLF, ACLF-SIRS, and ACLF-sepsis). **(F)** MitoTracker Green staining and **(G)** mitochondrial functionality based on MitoTracker Red vs. MitoTracker Green percentage in ACLF monocytes compared with healthy controls (*p < 0.05; **p < 0.01; ***p < 0.001; ****p < 0.0001).

Phagocytosis and oxidative burst function are energy-demanding processes. To understand the metabolic processes required for these functions, we inhibited glycolysis using 2-DG and OXPHOS using rotenone/antimycin and oligomycin and studied their effect on phagocytosis and oxidative burst in healthy monocytes. Treatment with rotenone/antimycin (*p* < 0.01), oligomycin (*p* < 0.01), and 2-DG (*p* < 0.05) significantly reduced the phagocytic capacity of healthy monocytes (**Figure 2A**). However, reduction in phagocytosis in response to inhibitors of mitochondrial respiration and glycolysis was comparable, suggesting that oxidative phosphorylation of glucose facilitates phagocytosis. Oxidative burst in response to *E. coli* was comparable between control, rotenone/antimycin A, and oligomycin; however, it was significantly (*p* < 0.001) reduced in monocytes treated with 2-DG (**Figure 2B**). Treatment of healthy monocytes with rotenone/antimycin, oligomycin, or 2-DG did not affect cell death, as assessed by annexin V staining (**Figure S2A**), indicating that anaerobic glycolysis is required to facilitate the oxidative burst function of monocytes.

To check the status of energy metabolism, we analyzed ACLF and healthy and cirrhotic monocytes for changes in glycolysis and OXPHOS. In comparison to healthy monocytes, there was a significant decrease in both OXPHOS (*p* < 0.01) and glycolysis (*p* < 0.0001) in cirrhotic monocytes. In comparison to cirrhosis, while OXPHOS (*p* < 0.001) further decreases in ACLF, glycolysis was comparable (**Figure 2C**), suggesting a broad defect in ACLF monocyte energy metabolism which becomes evident even at the stage of cirrhosis. Further analysis of different OXPHOS parameters showed a significant decrease in maximum respiration (MR, *p* < 0.0001) and ATP production (ATP, *p* < 0.001) between healthy and cirrhotic monocytes and in proton leak (PL, *p* < 0.001) between healthy and ACLF monocytes (**Figure S2B**), suggesting loss of mitochondrial function in ACLF monocytes, which is independent of ACLF etiology (**Figure S2C**).

Within the ACLF cohort, in comparison to ACLF, both ACLF-SIRS and ACLF-sepsis monocytes showed a significant reduction in basal, maximal, ATP, PL-linked OXPHOS (**Figures 2D**, **S2D**, **Table S2**), and glycolysis (**Figure 2E**, **Table S2**). Between ACLF-SIRS and ACLF-sepsis, while there was further reduction of basal (*p* = 0.05) and ATP (*p* < 0.05)-linked OXPHOS (**Figure 2D**, **Table S2**) in ACLF-sepsis, glycolysis was comparable (**Figure 2E**, **Table S2**). In further nominal logistic regression analysis, a progressive decrease in various OXPHOS parameters and glycolysis of ACLF monocytes were significantly (*p* < 0.05) associated with the development of SIRS and sepsis (**Table S3**), explaining the changes observed in ACLF monocyte phagocytosis and oxidative burst with the onset of SIRS and sepsis. The AUROC and cutoff values of various OXPHOS parameters and glycolysis for the onset of SIRS and sepsis are summarized in **Table S4**.

To investigate whether the loss of OXPHOS in ACLF monocytes resulted from the loss of mitochondrial mass or accumulation of dysfunctional mitochondria, we stained cells with MitoTracker Green (MTR, ΔΨm-independent mitochondrial stain) and MitoTracker Red CMXRos (MTR, ΔΨm-dependent mitochondrial stain). In comparison to control, ACLF monocytes showed a significant decrease (*p* < 0.001) in total mitochondrial mass (**Figure 2F**) with accumulation (*p* < 0.001) of dysfunctional mitochondria (MTG^+^MTR^−^) (**Figure 2G**). Together, these observations suggest that the loss of functional mitochondria leads to an OXPHOS defect that drives the defect in ACLF monocyte phagocytosis.

### Association of Systemic Inflammation and Endotoxemia With Defect in Monocyte OXPHOS

**Figure 3:**
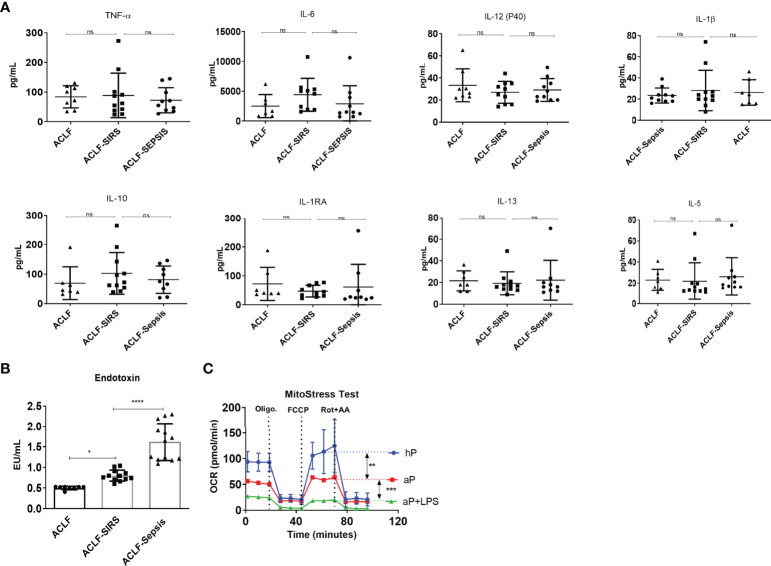
Association of systemic inflammation and endotoxemia with monocyte mitochondrial respiration defects in patients with ACLF. **(A)** Dot plot showing changes in plasma level of pro-inflammatory and anti-inflammatory cytokines [tumor necrosis factor-alpha (TNF-α); interleukins (IL), 6, 12, 1β, 10, 1RA, 13, and 5] in the ACLF subgroups (ACLF, ACLF-SIRS, and ACLF-sepsis). **(B)** Dot plot showing changes in plasma endotoxin levels in the ACLF subgroups (ACLF, ACLF-SIRS, and ACLF-sepsis). **(C)** Real-time changes in oxygen consumption rate (OCR) with subsequent treatment with oligomycin (Oligo.) FCCP and rotenone and antimycin A (Rot. + AA.) in healthy monocytes treated with healthy plasma (hP), ACLF plasma (aP), and ACLF plasma with LPS (aP + LPS) (*p < 0.05; **p < 0.01; ***p < 0.001; ****p < 0.0001). n.s. stands for non-significant.

Systemic endotoxemia and cytokine surge have been associated with ACLF and have shown to dampen monocyte phagocytosis (22). Next, to check how the plasma level of cytokines and endotoxin changes with the development of SIRS and sepsis in ACLF, we measured the levels of 24 different pro- and anti-inflammatory cytokines and endotoxin in the same patients. Interestingly, we observed that the plasma levels of all 24 different cytokines were comparable in ACLF, ACLF-SIRS, and ACLF-sepsis (**Figures 3A**, **S3**), while endotoxin significantly increases from ACLF to ACLF-SIRS (*p* < 0.05) and ACLF-SIRS to ACLF-sepsis (*p* < 0.0001) (**Figure 3B**).

To explore the impact of these factors on monocyte OXPHOS, we devised an *in-vitro* model of ACLF-mimic by culturing the freshly isolated healthy monocytes with pooled ACLF (no-SIRS, no-sepsis) plasma alone or with LPS for 6 h. Monocytes treated with healthy plasma were taken as control. Exposure of ACLF plasma alone significantly (*p* < 0.001) decreased the OXPHOS of monocytes, which was further dampened (*p* < 0.0001) in the presence of LPS (**Figure 3C**). Taken together, these data suggest that during ACLF, circulating plasma-derived factors suppress monocyte OXPHOS which are further dampened by systemic endotoxemia.

### Dynamic Assessment of Mitochondrial Function in ACLF Monocytes and Its Impact on Patient Outcome

We observed a significant decrease in monocyte OXPHOS from ACLF to ACLF-sepsis. To understand how it changes with the progression of ACLF, we followed the changes in monocyte OXPHOS till 72 h from the time of admission. Due to the limited availability of repeated blood samples, we were able to analyze kinetic changes (baseline, 24 h, and 72 h) in eight patients. Out of these eight ACLF patients, six had sepsis and two had no sepsis at the time of presentation. All patients received albumin; three patients also received plasma exchange together with albumin. With further therapeutic intervention, six (four with sepsis and two without sepsis) of eight patients showed improvement in monocyte OXPHOS, out of which one patient subsequently succumbed to COVID-19 and the remaining five recovered and were discharged. Two sepsis patients did not show any improvement in monocyte OXPHOS by 72 h and died within a week of admission (**Figure S4**).

**Figure 4:**
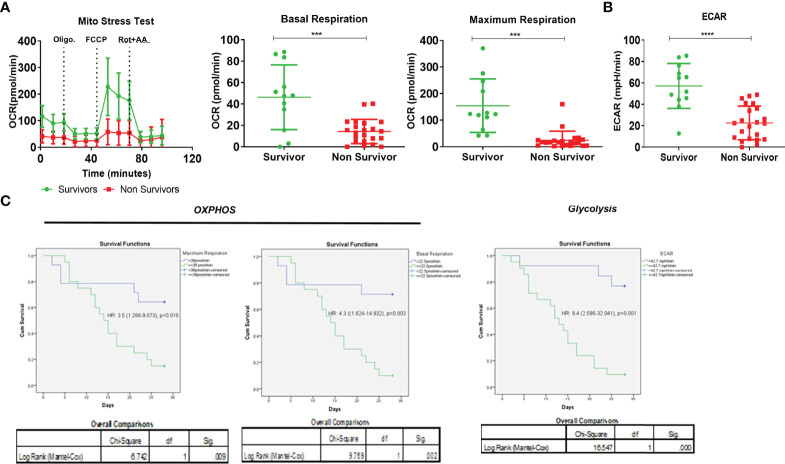
Effect of monocyte energy metabolism on ACLF patients’ outcomes. **(A)** Real-time changes in oxygen consumption rate (OCR) with subsequent treatment with oligomycin (Oligo.) FCCP and rotenone and antimycin A (Rot. + AA.) (left). Dot plot showing changes in basal mitochondrial respiration (middle) and maximum mitochondrial respiration (right) of monocytes in ACLF survivors and non-survivors. **(B)** Dot plot showing changes in glycolysis (extracellular acidification rate) of monocytes in ACLF survivors and non-survivors. **(C)** Kaplan–Meyer survivorship graph showing changes in monocyte maximal respiration, basal respiration, and glycolysis (extracellular acidification rate) in ACLF survivors vs. non-survivors (*p < 0.05; **p < 0.01; ***p < 0.001; ****p < 0.0001).

Out of the 34 ACLF patients in which monocyte bioenergetics was done at the time of admission, 22 died or underwent transplant (non-survivors) and 12 were alive (survivors) at 28 days of follow-up. In comparison to survivors, the monocytes of non-survivors showed a significant reduction in both OXPHOS [BR−, *p* < 0.001; MR, *p* < 0.001 (**Figure 4A**)] and glycolysis [*p* < 0.001 (**Figure 4B**)]. In the univariate Cox regression analysis, both glycolysis [hazard ratio (HR) = 9.1] and OXPHOS (BR, HR = 4.9; MR, HR = 3.5) significantly correlated with short-term (28 days) mortality in ACLF patients (**Table S5**). ACLF patients with monocyte glycolysis of ≤42.7 mpH/min (AUROC = 0.901, sensitivity 86.9%, specificity 83.3%) and with OXPHOS [BR ≤ 22.5 pmol/min (AUROC = 0.81, sensitivity 75%, specificity 77.3%); MR ≤ 37.9 pmol/min (AUROC = 0.82, sensitivity 77.3%, specificity 75%)] showed a significant increase in short-term mortality (**Figure 4C**). Together, these data suggest that loss of monocyte bioenergy adversely affects the outcome of ACLF patients.

### Umbilical Cord Mesenchymal Stem Cells and Monocyte Co-Culture Restore ACLF Monocyte Bioenergetics and Function

**Figure 5:**
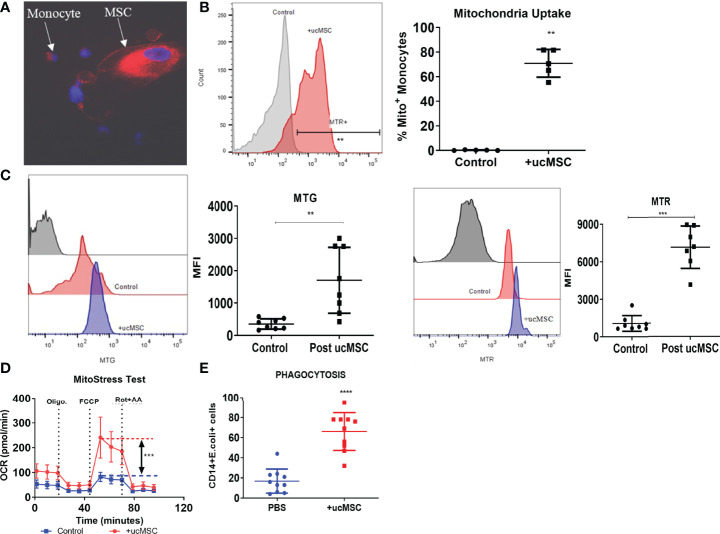
Effect of umbilical cord mesenchymal stem cells (ucMSCs) on ACLF monocyte bioenergy and function. **(A)** Representative immunofluorescence confocal image of MSC donating its mitochondria to the ACLF monocyte (×60). **(B)** Representative histogram (left) and dot plot (right) showing mitochondrial uptake by ACLF monocytes measured through flow cytometry. **(C)** Representative histograms and dot plot showing changes in MitoTracker Green (total mitochondria) and MitoTracker Red (functional mitochondria) intensity in ACLF monocytes co-cultured with ucMSCs. **(D)** Real-time changes in oxygen consumption rate (OCR) with subsequent treatment with oligomycin (Oligo.) FCCP and rotenone and antimycin A (Rot. + AA.) in ACLF patients’ monocytes co-cultured with ucMSC, compared with control. **(E)** Dot plot showing changes in phagocytic activity of ACLF monocytes co-cultured with ucMSCs compared with control (*p < 0.05; **p < 0.01; ***p < 0.001; ****p < 0.0001).

Our data showed that OXPHOS fuels monocyte phagocytosis and loss of total and functional mitochondria is associated with defective OXPHOS in ACLF monocytes. Recently, a number of studies have shown that mitochondrial transfer from MSCs improves the energy metabolism of various cells (38–40). We hypothesized that mitochondrial transfer from MSCs could restore ACLF monocyte OXPHOS and their phagocytosis. MSCs isolated from the umbilical cord showed characteristic fibroblast-like spindle-shaped morphology with >90% positive expression of MSC surface markers CD90, CD73, and CD105 and <1% expression of CD14/20/34/45 (**Figures S5A, B**). Passage 3 ucMSCs were labeled with MTR and co-cultured with ACLF monocytes. Using immunofluorescence imaging, we observed that after an overnight co-culture with ucMSCs, most of the ACLF monocytes acquire MTR^+^ MSC mitochondria (**Figure 5A**). Flow cytometry analysis showed that >70% (*p* < 0.01) of CD14^+^ ACLF monocytes were positive for MTR^+^ ucMSC mitochondria (**Figure 5B**), suggesting the transfer of mitochondria from ucMSC to ACLF monocytes. Post-ucMSC co-culture, there was a significant increase in both total (MTG^+^) (*p* < 0.01) and functional (MTG^+^MTR^+^) (*p* < 0.001) mitochondrial mass in ACLF monocytes (**Figure 5C**). Next, to evaluate whether this increase in functional mitochondrial mass can translate to an increase in ACLF monocyte energy and function, we determine the changes in monocyte OXPHOS and phagocytosis with and without ucMSCs. *In-vitro* overnight co-culture with ucMSCs significantly improved OXPHOS (*p* < 0.001; **Figure 5D**) and phagocytosis (*p* < 0.0001; **Figure 5E**) of ACLF monocytes. Together, these data showed that mitochondrial transfer from ucMSC restores ACLF monocyte OXPHOS and phagocytic function.

### ucMSC Therapy Resuscitates Monocyte Bioenergetics, Decreases Hepatocyte Injury, and Potentiates Regeneration in the ACLF Animal

**Figure 6:**
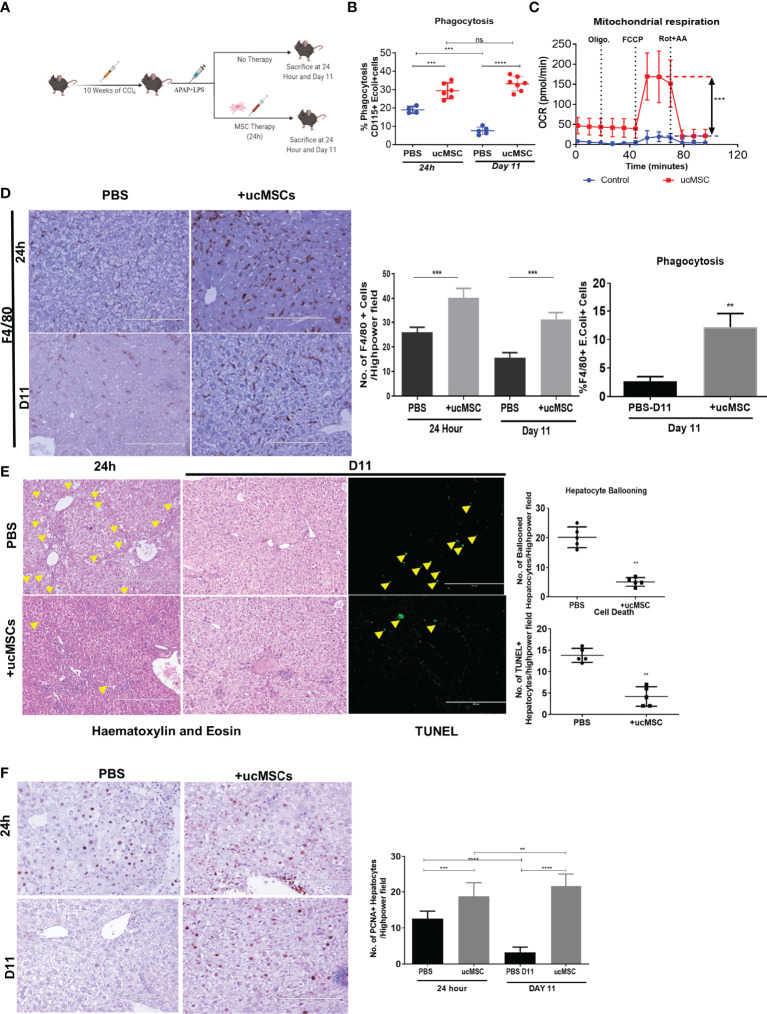
Effect of ucMSC therapy on monocyte energy and function, liver injury, and regeneration in the animal model of ACLF. **(A)** Schematic diagram of MSC therapy in the ACLF animal model. **(B)** Dot plot showing changes in CD115^+^ bone marrow monocyte phagocytosis at 24 h and day 11 in control and ucMSC-treated animals. **(C)** Real-time changes in oxygen consumption rate (OCR) with subsequent treatment with oligomycin (Oligo.) FCCP and rotenone and antimycin A (Rot. + AA.) in CD115^+^ bone marrow monocytes in control and ucMSC-treated animals. **(D)** Representative images showing F4/80^+^ liver macrophages in liver tissue sections (left) and bar graphs showing changes in the number of F4/80^+^ cells (middle) and F4/80^+^ liver macrophage phagocytosis (right) in control and ucMSC-treated animals at the given time points. **(E)** Representative images showing hematoxylin and eosin staining of liver tissue and TUNEL^+^ hepatocytes (left). Dot plot showing the number of ballooned hepatocytes and TUNEL^+^ hepatocytes (right) in control and ucMSC-treated animals at the given time points. **(F)** Representative images showing PCNA^+^ hepatocytes in liver tissue sections (left). Bar graph showing changes in the number of PCNA^+^ hepatocytes (right) in control and ucMSC-treated animals at the given time points (*p < 0.05; **p < 0.01; ***p < 0.001; ****p < 0.0001). n.s. stands for non-significant.

To check whether MSCs could restore monocyte energy and function *in vivo*, we analyzed the effect of MSC therapy on the change in monocyte phagocytosis, bioenergetics, and its impact on liver injury and regeneration in the animal model of ACLF. Firstly, we showed that human ucMSC infusion was well tolerated by healthy C57BL/6J mice, showing no changes in body weight (**Figure S6A**), AST, ALT (**Figure S6B**), and blood circulating lymphocytes (**Figure S6C**) and no histological change in the liver, kidney, and lung at 24 h or day 7 post-injection (**Figure S6D**). Next, we tested the therapeutic efficacy of ucMSC for restoring monocyte function in ACLF animals (**Figure 6A**). ucMSC-treated animals showed a significant increase in monocyte phagocytosis [24 h, *p* < 0.001; day 11, *p* < 0.0001 (**Figure 6B**)] and OXPHOS [24 h, *p* < 0.001 (**Figure 6C**)] compared with control. ucMSC-treated animals also showed a significant increase in the number of F4/80^+^ liver macrophages (24 h, *p* < 0.001; day 11, *p* < 0.001) and their phagocytosis (day 11, *p* < 0.01) compared with control (**Figure 6D**). This suggests that ucMSCs can resuscitate monocyte bioenergetics and prevent their dysfunction in ACLF. Further analysis of liver tissue showed a significant reduction in hepatocyte ballooning at 24 h, TUNEL^+^ hepatocyte, and fibrosis at day 11 in ucMSC-treated animals compared with control (**Figures 6E**, **S7**). They also showed a significant increase in the number of PCNA^+^ hepatocytes both at 24 h (*p* < 0.01) and day 11 (*p* < 0.001) in comparison with control. From 24 h to day 11 while control animals showed significant (*p* < 0.0001) loss of hepatocyte proliferation, animals with ucMSC therapy showed further increase in PCNA positivity (**Figure 6F**). These combined data suggest that ucMSC therapy not only rejuvenates monocyte bioenergetics and function but also reduces liver injury and potentiates native liver regeneration in the animal model of ACLF.

## Discussion

**Figure 7:**
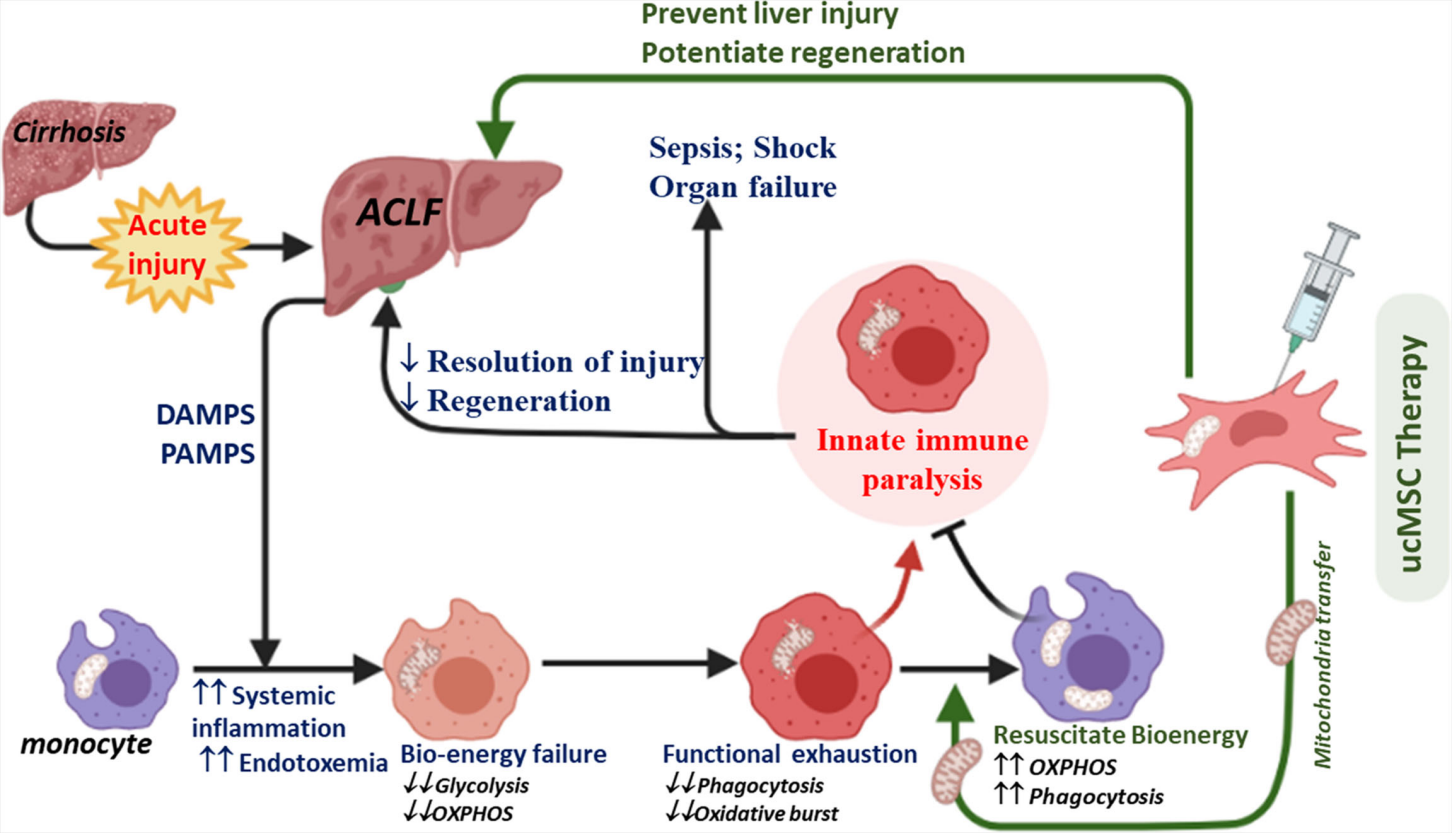
Diagram showing the proposed mechanism of ucMSC therapy in ACLF. Increased systemic accumulation of DAMPS, PAMPS, and inflammatory mediators in response to acute liver injury in cirrhosis (a condition called ACLF) induces mitochondrial damage and bioenergy failure in monocytes. A broad defect in energy metabolism drives the exhaustion of phagocytic and oxidative burst function of monocytes in ACLF, required for the effective clearance of pathogens and cellular debris. This may account for the poor resolution of liver injury and infection, leading to regeneration failure, sepsis, shock, and death in ACLF. ucMSCs rejuvenate monocyte energy and function, prevent liver injury, and potentiate regeneration in ACLF animals.

Aggravated systemic inflammatory and oxidative stresses due to uncontrolled liver injury and poor infection control are associated with regeneration failure, multi-organ dysfunction, septic shock, and death in ACLF (34, 36, 41). The underlying mechanisms of the host’s innate immune failure to resolve liver injury and infection in ACLF are partially understood. Here, we demonstrate that bioenergetic failure drives the functional exhaustion of monocytes in ACLF. We further demonstrate the importance of monocyte bioenergetics in the spontaneous recovery of ACLF and show that rejuvenating monocyte bioenergetics by ucMSC improves monocyte phagocytic function, prevents liver injury, and potentiates regeneration in ACLF (**Figure 7**).

Liver macrophages together with monocytes play an important role in the host’s innate immune defense (13–16). In our study, we observed that while the number of monocytes–macrophages in the liver and peripheral blood increased in ACLF, they displayed profound defects in *ex-vivo* phagocytic and oxidative burst capacity. To understand the underlying defects, we focused on the energy metabolism of monocytes and showed that while OXPHOS fuels the phagocytosis, glycolysis fuels the oxidative burst function of monocytes. In comparison to healthy monocytes, ACLF monocytes showed significant defects in glycolysis and OXPHOS with loss of total and functional mitochondria, suggesting that broad defects in monocyte energy metabolism drive the loss of monocyte phagocytic and oxidative burst function in ACLF.

During ACLF, acute insult in chronic liver injury leads to the development of SIRS and the subsequent progression of secondary organ dysfunction and/or sepsis (9). In comparison to healthy and cirrhotic monocytes, ACLF showed a marked increase in plasma levels of various inflammatory and anti-inflammatory cytokines (8, 42), which is thought to induce innate immune dysfunction in ACLF. However, in ACLF, it is unclear whether patients succumb to infection due to failure of monocytes/macrophages to effectively clear the invading pathogen leading to sepsis and organ failure or whether prolonged anti-inflammatory response renders the patient incapable to respond to secondary infections. Our data showed a significant reduction in monocyte bioenergetics and its phagocytic and oxidative burst function in cirrhosis which were further compromised in ACLF and worsened with the onset of SIRS and sepsis. In this study, we observed that while the plasma levels of pro- and anti-inflammatory cytokines were comparable, there was a significant increase in endotoxin from ACLF to ACLF-SIRS and ACLF-SIRS to ACLF-sepsis. We further demonstrated that treatment of healthy monocytes with ACLF plasma significantly reduced OXPHOS which was further dampened with LPS, suggesting the potential role of systemic inflammation and endotoxemia in bioenergetic suppression of monocytes in ACLF. Previous studies showed that increased prostaglandin E2 (PGE2) and EP2-mediated signaling in response to acute injury dampened the monocyte–macrophage function (43, 44) and drove the immunosuppression in ACLF (20). Recently, PGE2–EP2 signaling has been shown to dampen both glycolysis and OXPHOS of macrophages by reducing the glucose flux and mitochondrial respiration (45). Indeed, patients with ACLF have shown an increase in glucose flux to pentose and D-glucuronate pathways (46). Similarly, LPS-induced suppression of *de-novo* NAD synthesis dampens the macrophage OXPHOS and phagocytosis in a dose-dependent manner (47), leading to immune paralysis in sepsis (33). Taken together, it is tempting to speculate that a profound increase in plasma inflammatory mediators like PGE2 in response to acute liver injury may lead to bioenergetic suppression of ACLF monocytes. This results in the maladaptive immune response against infection and a rise in systemic endotoxemia which further drives the loss of monocyte bioenergy and function during the course of ACLF. This may account for the poor resolution of injury and infection, leading to sepsis, secondary organ failure, shock, and death in ACLF. Indeed, our data showed that the loss of monocyte bioenergetics adversely affects the ACLF outcome. There was a progressive improvement in monocyte OXPHOS in patients with spontaneous recovery; however, patients who failed to improve showed early mortality. Recently, we have shown that ACLF patients that fail to improve monocyte energy and function do not respond to plasma exchange therapy even with the dampening of cytokines and endotoxin (48). This further highlights the clinical importance of monocyte energy metabolism and the need for a therapeutic strategy that can rejuvenate the monocyte energy metabolism in ACLF.

MSCs have been shown to improve the energy metabolism of various cells including monocytes–macrophages by donating their mitochondria (38–40). ACLF patients showed a significant loss of mitochondrial mass and function; hence, we investigated whether ucMSCs could resuscitate ACLF monocyte bioenergetics and phagocytosis. *In-vitro* co-culture with ucMSCs significantly improved the monocyte OXPHOS and phagocytic function. ucMSCs transferred their mitochondria and resuscitated the functional mitochondrial mass of monocytes. Further ucMSC therapy in ACLF animals showed improvement of monocyte energy and phagocytosis. Hence, ucMSCs can be used to resuscitate monocyte energy and phagocytic function in ACLF. Interestingly, similar to ACLF, patients with late-stage sepsis also show defects in mitochondrial energy metabolism in monocytes (33), and the use of MSCs has shown promising results in the practical model of sepsis (49). MSC therapy has also been shown to be safe and improves the outcome in ACLF (50, 51). Our data also showed significant reduction in liver injury and improvement of hepatocyte regeneration with ucMSC therapy. However, our data do not explain whether improvement in liver injury and regeneration in ACLF animals are due to improved monocyte energy and function or other immunomodulatory and paracrine functions of MSCs. Supplementing and resolving macrophage function in acute liver injury have recently been shown to improve the resolution of liver injury and regeneration (52), and we observed a significant increase in liver macrophage number and their phagocytic function with ucMSC therapy. Hence, improved monocyte energy and function may contribute to the observed reduction of liver injury and improvement of regeneration in ACLF animals.

In conclusion, broad defects in energy metabolism drive the exhaustion of phagocytic and oxidative burst function of ACLF monocytes. Our study provides new insight into the pathophysiology of innate immune dysfunction in ACLF along with evidence that approaches like MSC therapy can correct this problem. This also highlights the importance of bioenergetic rejuvenating therapeutic strategies in the treatment of ACLF. It will be intriguing for future work to further fine-tune and develop this approach as a potential interventional strategy to treat and/or prevent immune dysfunction in ACLF.

## Data Availability Statement

The raw data supporting the conclusions of this article will be made available by the authors, without undue reservation.

## Ethics Statement

The studies involving human participants were reviewed and approved by the Institutional Ethics Committee. The patients/participants provided their written informed consent to participate in this study. The animal study was reviewed and approved by the Institutional Animal Ethics Committee, Institute of Liver and Biliary Sciences.

## Author Contributions

AK and SKS contributed to the design of the studies. DM and DK performed most of the experiments, with the assistance from RJ, NN, RK, AH, and RS. GK performed the statistical analysis. CB helped in the histopathology analysis. SS recruited patients for umbilical cord samples. RM, SKS, and RK were responsible for the recruitment, diagnosis, and clinical management of ACLF patients. VP provided the explant ACLF and healthy human liver. MB provided the healthy donor blood. NT and SS helped in the FACS analysis. AK, RM, and SKS assisted with the interpretation of the findings. The manuscript was written by AK and DM with critical input from SKS and RM. All authors contributed to the article and approved the submitted version.

## Funding

This work was supported by the Science and Engineering Research Board (SERB-DST), Government of India (Grant IR/SB/EF/02/2016), and the Department of Biotechnology, Government of India (Grant BT/PR21543/MED/31/351/2016).

## Conflict of Interest

The authors declare that the research was conducted in the absence of any commercial or financial relationships that could be construed as a potential conflict of interest.

## Publisher’s Note

All claims expressed in this article are solely those of the authors and do not necessarily represent those of their affiliated organizations, or those of the publisher, the editors and the reviewers. Any product that may be evaluated in this article, or claim that may be made by its manufacturer, is not guaranteed or endorsed by the publisher.

## Abbreviations

ACLF, acute-on-chronic liver failure; ucMSCs, umbilical cord mesenchymal stem cells; CCl_4_, carbon tetrachloride; APAP, acetaminophen; LPS, lipopolysaccharide; OCR, oxygen consumption rate; ECAR, extracellular acidification rate; SIRS, systemic inflammatory response; TUNEL, terminal deoxynucleotidyl transferase; APASL, Asian Pacific Association for the Study of the Liver; AARC, APASL ACLF Research Consortium; AUROC, area under the receiver operating characteristic curve; BM, bone marrow; HLA-DR, human leucocyte antigen-DR; TLR, toll-like receptor; TCA, tricarboxylic acid cycle; ROS, reactive oxygen species; MSC, mesenchymal stem cell; INR, international normalized ratio; LDLT, live donor liver transplant; OXPHOS, oxidative phosphorylation; FCCP, fluorocarbonyl cyanide phenylhydrazone; 2-DG, 2-deoxy-D-glucose; MR, maximum respiration; MTG, MitoTracker Green; MTR, MitoTracker Red CMXRos; PGE2, prostaglandin E2.
