# Supplementary material for: Bioenergetic Failure Drives Functional Exhaustion of Monocytes in Acute-on-Chronic Liver Failure
Source: Front Immunol. 2022 Jun 3;13:856587. doi: 10.3389/fimmu.2022.856587 (PMC9210982; doi:10.3389/fimmu.2022.856587)
Supplement: Supplementary file 1 [file DataSheet_1.docx]

**Supplementary Material**

**Bioenergetic failure drives functional exhaustion of monocyte in Acute-on-Chronic Liver Failure**

Deepanshu Maheshwari^1†^, Dhananjay Kumar^1†^, Rakesh Kumar Jagdish^2^, Nidhi Nautiyal^1^, Ashinikumar Hidam^1^, Rekha Kumari^1^, Rashi Sehgal^1^, Nirupama Trehanpati^1^, Sukriti Sukriti^1^, Guresh Kumar^1^, Swati Sinha^6^, Meenu Bajpai^3^, Viniyendra Pamecha^4^, Chhagan Bihari^5^, Rakhi Maiwall^2^, Shiv K Sarin^2*^, Anupam Kumar^1*^

**Supplemental methods:**

**Phenotypic analysis of monocyte and liver macrophages**

Peripheral blood mononuclear cells (PBMCs) were isolated and stained with the following antibodies: CD14, CD16, (eBioscience, San Diego, California, USA) and matching isotype controls. For analysis of liver macrophage liver tissue were subjected to enzymatic digestion (collagenase IV), parenchymal and non-parenchymal cells were fractionated by density gradient centrifugation. Isolated non-parenchymal cells were stained with CD68, CD14, CD16 (eBioscience, San Diego, California, USA). Flow cytometric data acquisition was performed on BD FACSAria and analysed using FlowJo™ v10.6.2.

**Isolation, culture and characterization of umbilical cord mesenchymal stem cells**

Umbilical cords (gestational ages 31 to 37 weeks) were obtained from the maternity hospitals after normal or caesarean deliveries. The cords were collected after obtaining informed consent from the mother. Approximately 10 cm of the cord was collected into tubes containing PBS supplemented with penicillin and streptomycin and were transported to the lab within 24 hours of delivery. Processing was done in a GMP compliant environment. The Cords were disinfected with 1% povidone-iodine solutions, followed by washing with phosphate buffered saline (PBS) supplemented with penicillin and streptomycin. Disinfected cords were dissected, and blood vessels were removed, cords were cut into 2 cm^2^ pieces and were placed in 6 well plates. Tissues were cultured in MSC-Brew GMP medium (Miltenyi Biotec, 170-076-325) containing 10% Human Platelet Lysate (STEMCELL technology) and incubated at 37C in 5% CO2 undisturbed for 4-5 days after which fresh media were added every 5th day, Tissue explants were removed after 3 weeks of culture, adherent cells were passaged and reseeded for further expansion when adherent cells reached 90% of confluency. Cells were passaged with TrypLE Select enzyme (Gibco, New York, USA). Approximately 2×10^6^ cells of passage-3 were further used for characterization of MSC marker phenotypes using fluorescently antibody (CD90, CD73, CD105, CD14/20/34/45). After incubation with antibody for 20 mins at 37C labelled cells were washed FACS staining solution (PBS with 2% FBS) and resuspended in staining solution having 0.1% paraformaldehyde, samples were analysed using BD FACSVerse cytometer, data was analysed using FlowJo™ v10.6.2.

**Monocyte phagocytic and oxidative burst capacity following exposure to Escherichia coli:** The phagocytic capacity of monocytes was assessed using Phagocytosis Assay Kit (*E.coli*- FITC inactivated ) (Cayman Chemical) Briefly, 1x10^5^ purified monocytes were incubated in RPMI media with *E. coli* FITC complex and incubated at 37C for 40 minutes, cells were washed with assay buffer and read on flow cytometer. For respiratory burst, 2x10^5^ monocytes were first incubated with 10X -Dihydro-rhodamine-123 (DHR-123) for 15 minutes at 37C followed by incubation with LPS (100ng/ml) for 2hour. Cells were washed with assay buffer and resuspended in 0.5mL assay buffer and immediately read on flow cytometer. Data was analysed using FlowJo™ v10.6.2.

**Mitochondrial biomass and membrane potential:** Mitochondrial biomass and membrane potential of purified monocytes was assessed using MitoTracker Green FM (ThermoFisher Scientific, M7514) and MitoTracker Red FM (ThermoFisher Scientific, M22425). Cells were incubated with MitoTracker dyes for 30 minutes at room temperature after which they were immediately read on Flow Cytometer. Data was assessed using FlowJo™ v10.6.2.

**Evaluation of Histopathology**

The tissues were ﬁxed in 10% paraformaldehyde solution and processed using parafﬁn block techniques in wax, deparafﬁnization in xylene, and dehydration in alcohol. Tissues were sectioned at >2.5µm and stained with hematoxylin and eosin (H&E) as per the standard protocols to study the change in histology. Liver pathology was evaluated in a blind manner by the pathologist. Sections were examined using EVOS FL light microscopy and quantitative analysis was performed using ImageJ in three non-overlapping random fields at 200× magnification.

**Immunohistochemistry**

Paraffin-embedded liver sections (>2.5 μm thick) were stained with primary antibodies as follows: Rabbit-anti-Proliferating Cell Nuclear Antigen (PCNA; Cloud-Clone Corporation, Polyclonal Antibody (PA), Texas, USA; 1:200), F4/80 (Cell Signalling Technology, Massachusetts, USA; 1:200) Secondary Antibody purchased from Vector laboratories (ImmPRESS HRP Anti-Rabbit IgG (Peroxidase) Polymer Detection Kit, made in Horse and ImmPRESS HRP Anti-Mouse IgG (Peroxidase) Polymer Detection Kit, made in Goat) was used. DAB and Substrated chromogen (Vector Lab.), diluted in 1:1 ratio, consisting of streptavidin-horseradish peroxidase and 3,3'-diaminobenzidine (DAB) substrate was employed for visualization. Quantitative analysis was performed using ImageJ at different random fields

**TUNEL Assay:**

Cell death was studied using in situ labeling of apoptosis-induced DNA strand breaks (TUNEL assay) using the In Situ Cell Death Detection Kit (Roche; CAT No. 11684795910) as per the manufacturer’s protocol.

**Endotoxin Estimation:**

Plasma endotoxin levels were also measured in the same group of patients using limulus amoebocyte lysate (LAL) assay, a chromogenic quantification assay (Pierce Chromogenic Endotoxin Quant Kit, Thermo Scientific) according to the manufacturer’s protocol. Briefly, samples were mixed with the LAL supplied in the test kit and incubated at 37 ± 1°C for 10 minutes. Substrate solution was then mixed with the LAL-sample and incubated at 37 ± 1°C for an additional 6 minutes. The reaction was stopped with stop solution. The absorbance of the sample was determined spectrophotometrically at 410 nm. Because this absorbance was in direct proportion to the amount of endotoxin present, the concentration of endotoxin was calculated from a standard curve. The data were represented with the arbitrary unit EU/mL.

**Plasma cytokine measurement:**

Milliplex MAP Human Cytokine/Chemokine Magnetic Bead panel- premixed 21-plex Immunology Multiplex assay (TNF-α, IL-6, IL-12 (P40), IL-10, IL-1RA, IL-13, IL-5, RANTES, IL-17, IL-15, IL-17A, IL-2, TNF-β, MIP-1B, IL-12 (P70), G-CSF, GM-CSF, EGF, VEGF) were used to determine plasma cytokines. All measurements were done in duplicates according to manufacturer’s protocol. Briefly, 50 μL of microparticles precoated with specific antibodies were added to each well with standard or 25 μL of plasma samples and incubated for 60 minutes at room temperature in the dark. After washing the plate, 25 μL of detection antibody solution was added and the plate was incubated for 30 minutes at room temperature in the dark. A mix with streptavidin-PE solution was added to the plate for 30 minutes, and then the median relative fluorescence units from the antibody reactions was measured in 120 μL of reading buffer using a Luminex 200 analyzer (Luminex, Austin, TX) and the xPONENT software (v. 3.1; Luminex).

**Mouse models**

Chronic liver injury was induced by intraperitoneal administration of carbon tetrachloride (CCl4; Central Drug House, Delhi, India) in Olive oil (HiMedia Pvt. Ltd., India). We used an increasing dose of CCl4 approach to develop chronic liver injury. Chronic liver fibrosis was induced in 6–8 weeks male C57BL6 mice over a 10 weeks period by twice weekly intraperitoneal injections of CCl4 dissolved in sterile olive oil at a concentration of 0.1 ml/kg for first 3 weeks increasing to 0.2 m/kg for next 3 weeks followed by 0.5 ml/kg of CCl4 till they developed grade 3/4 fibrosis by week 10. After 10 week of chronic liver injury, ACLF injury were induced by signal dose acetaminophen (APAP) and lipopolysaccharide (LPS). To develop ACLF injury, mice were fasted for 12 h before administrating APAP (350 mg/kg, i.p) followed by LPS (50 µg/kg, i.p) 60 min post-APAP.

**ucMSC administration in animals.**

To study the tolerability of human ucMSC to immunocompetent mice, healthy 8–10-week-old C57Bl6 mice (n=20) were used. Animals were randomly divided into two groups. Group-1 received signal dose of ucMSC (1x10^6^/kg body weight) resuspended in PBS through tail vein injection. In group-2 PBS alone (100ul)through tail vain were infused as control. Animals were humanly sacrificed at 24hour and day 7 post MSC infusion, blood and organs were collected for biochemical, immunological and histological parameter respectively. To study the therapeutic potential of ucMSC 24 C57Bl6 mice with ACLF injury were used. 16-hour post ACLF injury, animals were divided into two groups. While group-1 received ucMSC (1x10^6^/kg body weight) resuspended in PBS via tail vain, group-2 received PBS as control. Mice were euthanised by overdose of ketamine post- 24 hours and day 11 of cell therapy, blood was collected through retro-orbital bleeding, liver were harvested for cells isolation and histology. Statistical analysis for animal experiment was performed in Graphpad Prism 6. Results are represented as mean±SD. Comparison between more than two groups was performed using ANOVA with post-hoc Turkey correction for multiple comparisons. When only 2 groups were compared an unpaired Student’s t test was used. Statistical significance is indicated in figures as *, p<0.05; **, p<0.01; ***, p<0.001; ****, p<0.0001.

**Preparation of stock solution**

Stock solutions of Oligomycin (6.32mM), FCCP (10mM), Rotenone(500mM) and Antimycin A (3.645mM) were prepared in DMSO. Working concentrations of oligomycin (1uM), FCCP (0.75uM), Rotenone (0.5uM) and Antimycin A (0.5uM) were prepared in incomplete RPMI. Deoxy-2-Glucose (2-DG) (304.58mM) stock solution was prepared in distilled water. Working concentration of 2-DG (50mM) was prepared in incomplete RPMI.

**Supplementary Table 1:** Demographic and **Clinical characteristics of patients admitted with ACLF, ACLF-SIRS and ACLF-sepsis**

| Clinical Parameter | ACLF- (n=8) | ACLF-SIRS(n=13) | ACLF-Sepsis(n=13) | p Value | p Value ACLF vs ACLF-SIRS | p Value ACLF -SIRS vs ACLF-Sepsis |
| --- | --- | --- | --- | --- | --- | --- |
| Age (years) | 43.38±6.71 | 42.08±10.88 | 42.38±8.86 | 0.27 | 0.426 | 1.000 |
| Gender, Male (%) | (8 (100%)) | (13 (100%)) | (11 (84.61)) | 0.33 | - | - |
| Aetiology (Acute)  *Alcoholic*  *Viral*  *DILI* | 4 (50%)  3 (37.5%)  1 (12.5%) | 9 (69.23%)  3 (23.07%)  1 (7.69%) | 9 (69.23%)  2 (15.38%)  2 (15.38%) |  |  |  |
| Temperature (°F) | 98.3±0.185 | 98.33±0.15 | 99.20±1.32 | 0.02 | 1.000 | 0.040 |
| Heart Rate (BPM) | 83.5±3.81 | 98.31±14.58 | 97.92±17.05 | 0.05 | 0.077 | 1.000 |
| Respiratory Rate | 19.75±0.70 | 21.08±1.75 | 22.15±2.23 | 0.02 | 0.331 | 0.410 |
| Lactate (mmol/l) | 1.18±0.46 | 2.13±0.61 | 2.68±1.76 | 0.02 | 0.250 | 0.744 |
| Total Bilirubin (mg/dl) | 17.88±10.47 | 25.59±9.88 | 21.35±8.02 | 0.18 | 0.229 | 0.771 |
| ALT (U/L) | 183.63±244.27 | 87.54±74.20 | 88.38±100.02 | 0.25 | 0.407 | 1.000 |
| AST (U/L) | 396.13±452.52 | 174.08±62.15 | 123.38±72.36 | 0.03 | 0.103 | 1.000 |
| ALP (U/L) | 109.13±46.87 | 108.15±43.00 | 99.46±47.24 | 0.85 | 1.000 | 1.000 |
| GGT (U/L) | 150.63±152.14 | 106.62±139.60 | 48.92±39.98 | 0.15 | 1.000 | 0.639 |
| Albumin (g/dl) | 2.35±0.29 | 2.30±0.33 | 2.45±0.20 | 0.38 | 1.000 | 0.524 |
| Creatinine (mg/dl) | 0.61±0.20 | 1.46±1.29 | 1.32±1.16 | 0.21 | 0.274 | 1.000 |
| Urea (mg/dl) | 31.43±25.92 | 52.52±33.94 | 52.10±45.72 | 0.39 | 0.661 | 1.000 |
| INR | 2.06±0.47 | 2.55±1.10 | 2.32±0.73 | 0.45 | 0.638 | 1.000 |
| PCT (ng/mL) | 0.91±1.18 | 1.51±1.82 | 3.11±4.07 | 0.18 | 1.000 | 0.478 |
| CRP (mg/L) | 24.49±34.93 | 28.96±30.97 | 62.51±60.35 | 0.10 | 1.000 | 0.206 |
| MAP (mm Hg) | 82.38±5.60 | 80.08±8.07 | 77.31±12.20 | 0.48 | 1.000 | 1.000 |
| Haemoglobin (gm/dl) | 10.65±2.58 | 10.98±1.38 | 11.30±2.28 | 0.77 | 1.000 | 1.000 |
| Total Leukocyte Count (x10^9^/L) | 7.86±2.15 | 14.54±5.42 | 13.22±10.35 | 0.13 | 0.155 | 1.000 |
| Platelet count (x10^9^/L) | 139.38±79.61 | 146.15±57.50 | 119.77±58.22 | 0.55 | 1.000 | 0.891 |
| Neutrophils (x10^9^/L) | 65.50±8.40 | 79.10±11.08 | 76.20±13.37 | 0.03 | 0.040 | 1.000 |
| Lymphocytes (x10^9^/L) | 20.20±9.04 | 11.70±9.17 | 11.17±9.56 | 0.08 | 0.152 | 1.000 |
| Culture Positive  Infiltrates on Chest X-Ray  Cellulitis |  |  | 7  4  2 |  |  |  |
| MELD | 25±2.39 | 31.23±6.36 | 29.46±5.69 | 0.05 | 0.048 | 1.000 |
| Number of organ failure | 1.00±0 | 2.14±1.51 | 3.57±1.45 | 0.001 | 0.08 | 0.01 |
| Child Pugh Score | 11±1.685 | 11.46±1.664 | 11.15±1.676 | 0.35 | 0.475 | 1.000 |
| AARC Score | 7.38±1.06 | 10.08±1.84 | 9.54±2.90 | 0.03 | 0.031 | 1.000 |
| Ascites Grade | 1 (42.86%)  2 (57.14%)  3 (28.57%) | 1 (7.69%)  2 (76.92%)  3 (15.38%) | 1 (0.00%)  2 (76.92%)  3 (23.08%) |  |  |  |
| 28 days transplant free survival | 6 (75%) | 4 (30.76%) | 2 (15.38%) |  |  |  |

**Supplementary Table 2: Comparison of monocyte respiratory (Mitochondrial and Glycolysis) Parameters in ACLF, ACLF-SIRS and ACLF-Sepsis**

| Parameter | ACLF (Mean±SD) | ACLF-SIRS (Mean±SD) | ACLF-Sepsis (Mean±SD) | p Value | p value | | |
| --- | --- | --- | --- | --- | --- | --- | --- |
|  |  |  |  |  | No SIRS vs SIRS | No SIRS vs SIRS | SIRS vs Sepsis |
| Basal Respiration (pmol/min) | 61.22±21.75 | 23.23±13.28 | 8.47±6.04 | <0.001 | <0.001 | <0.001 | 0.05 |
| Maximal Respiration (pmol/min) | 195.02±100.35 | 52.64±43.83 | 11.27±8.23 | <0.001 | <0.001 | <0.001 | 0.196 |
| Proton Leak (pmol/min) | 14.82±7.69 | 4.36±5.31 | 3.30±3.32 | <0.001 | <0.001 | <0.001 | 1 |
| ATP Production (pmol/min) | 46.393±19.59 | 19.48±8.26 | 6.56±6.45 | <0.001 | <0.001 | <0.001 | 0.02 |
| Spare Reserve Capacity (pmol/min) | 133.80±81.16 | 37.51±34.85 | 5.86±11.50 | <0.001 | <0.001 | <0.001 | 0.245 |
| ECAR (mph/min) | 65.96±16.88 | 32.61±14.43 | 17.69±16.26 | <0.001 | <0.001 | <0.001 | 1 |

**Supplementary Table 3: Nominal Logistic Regression Analysis showing association of monocyte respiratory (Mitochondrial and Glycolysis) parameters with development of SIRS and Sepsis in AACLF**

| Parameter | Group | Odd Ratio (95 CL) | P value |
| --- | --- | --- | --- |
| Basal Respiration (pmol/min) | ACLF | 1 |  |
|  | ACLF-SIRS | 0.844 (0.716-0.996) | 0.044 |
|  | ACLF-Sepsis | 0.733 (0.597-0.901) | 0.003 |
| Maximal Respiration (pmol/min) | ACLF | 1 |  |
|  | ACLF-SIRS | 0.967 (0.938-0.996) | 0.028 |
|  | ACLF-Sepsis | 0.834 (0.733-0.949) | 0.006 |
| Proton Leak (pmol/min) | ACLF | 1 |  |
|  | ACLF-SIRS | 0.782 (0.643-0.952) | 0.014 |
|  | ACLF-Sepsis | 0.735 (0.584-0.924) | 0.008 |
| ATP Production (pmol/min) | ACLF | 1 |  |
|  | ACLF-SIRS | 0.788 (0.627-0.990) | 0.041 |
|  | ACLF-Sepsis | 0.631 (0.476-0.837) | 0.001 |
| Spare Reserve Capacity(pmol/min) | ACLF | 1 |  |
|  | ACLF-SIRS | 0.965 (0.935-0.997) | 0.030 |
|  | ACLF-Sepsis | 0.899 (0.832-0.972) | 0.007 |
| ECAR (mph/min) | ACLF | 1 |  |
|  | ACLF-SIRS | 0.861 (0.752-0.985) | 0.030 |
|  | ACLF-Sepsis | 0.809 (0.699-0.938) | 0.005 |

**Supplementary Table 4a: AUROC analysis showing cut-off of monocyte respiratory (Mitochondrial and Glycolysis) parameters for diagnosis of SIRS in ACLF**

|  | |  | |  | |  | |  | |  | |  | |
| --- | --- | --- | --- | --- | --- | --- | --- | --- | --- | --- | --- | --- | --- |
| Parameter | | Cut-off | | AUC (%) | | Sensitivity (%) | | Specificity (%) | | Youden Index (%) | |  |  |
| ATP (pmol/min) | | <32.13674 | | 94.2 | | 100 | | 87.5 | | 87.5 | |  |  |
| Basal Respiration (pmol/min) | | <40.08 | | 74.0 | | 84.16 | | 87.5 | | 71.66 | |  |  |
| ECAR (mph/min) | | <47.34 | | 96.2 | | 61.5 | | 62.5 | | 24 | |  |  |
| Maximal Respiration (pmol/min) | | <114.4342 | | 91.3 | | 76.9 | | 75 | | 51.9 | |  |  |
| Proton Leak (pmol/min) | | <7.614086 | | 88.5 | | 76.9 | | 75 | | 51.9 | |  |  |
| Spare Reserve Capacity (pmol/min) | | <77.47846 | | 86.5 | | 76.9 | | 62.5 | | 39.4 | |  |  |

**Supplementary Table 4b: AUROC analysis showing cut-off of monocyte respiratory (Mitochondrial and Glycolysis) parameters for diagnosis of Sepsis in ACLF**

| Parameter | Cut-off | AUC (%) | Sensitivity (%) | Specificity (%) | Youden Index (%) |
| --- | --- | --- | --- | --- | --- |
| ATP (pmol/min) | <20.49129 | 99 | 92.3 | 87.5 | 79.8 |
| Basal Respiration (pmol/min) | <20.7 | 94.2 | 84.6 | 87.5 | 72.1 |
| ECAR(mph/min) | <41.8 | 97.1 | 84.6 | 87.5 | 72.1 |
| Maximal Respiration (pmol/min) | <40.04485 | 100 | 100 | 100 | 100 |
| Proton Leak (pmol/min) | <6.753089 | 94.2 | 92.3 | 87.5 | 79.8 |
| Spare Reserve Capacity (pmol/min) | <35.1632 | 99 | 92.3 | 87.5 | 79.8 |

**Supplementary Table 5: Univariant cox regression and AUROC analysis showing association of monocyte respiratory (Mitochondrial and Glycolysis) parameters with 28 days mortality in ACLF**

| Parameter | Cut off  (pmol/min) | AUC  (%) | Sensitivity  (%) | Specificity  (%) | Hazard Ratio  (%) |
| --- | --- | --- | --- | --- | --- |
| ATP (pmol/min) | <19.5 | 77.9 | 72 | 75 | 3.35 |
| Basal Respiration(pmol/min) | <22.5 | 81 | 75 | 77.3 | 4.9 |
| ECAR (mph/min) | <42.7 | 90.1 | 86.9 | 83.3 | 9.4 |
| Maximal Respiration(pmol/min) | <37.9 | 82 | 77.3 | 75 | 3.5 |
| Spare Reserve Capacity(pmol/min) | <22.8 | 78.8 | 77.3 | 75 | 3.5 |

**Supplemental Figures**

**
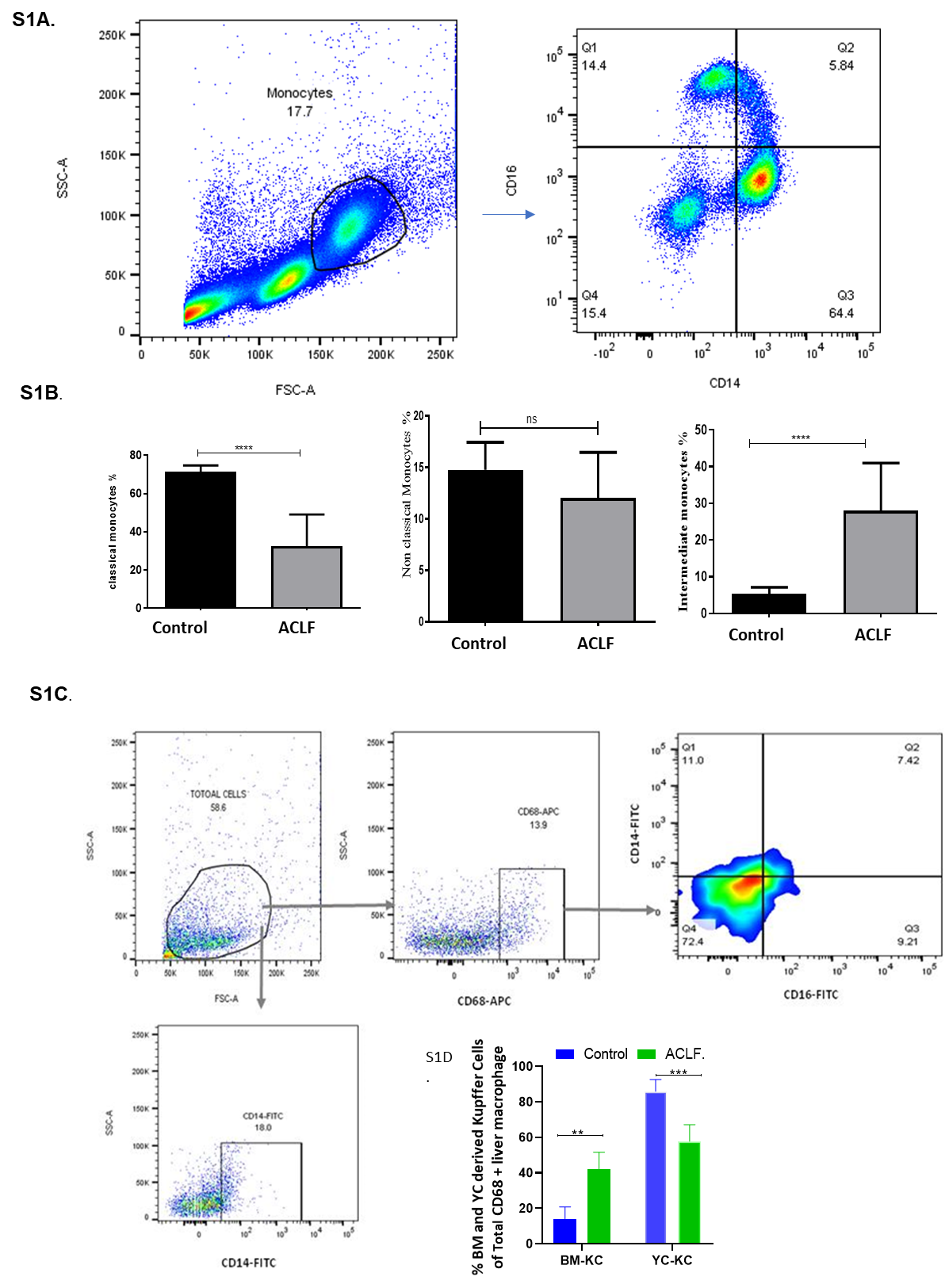
**

**Figure S1:** (S1A-B)-(A) Diagram showing flowcytometric gating strategy for identification of monocytes subsets in peripheral blood mononuclear cells. (B) Bar graphs showing changes in peripheral blood monocytes subsets (Classical, Non-Classical and Intermediate) in ACLF vs. Control. (C) Diagram showing flowcytometric gating strategy for identification of Kupffer Cells in Liver Infiltrating Lymphocytes (LIL), (D) Bar graphs showing changes in Bone Marrow Derived (BM-KC) and Yolk-Sac Derived (YC-KC) Kupffer Cells in ACLF vs healthy control

**
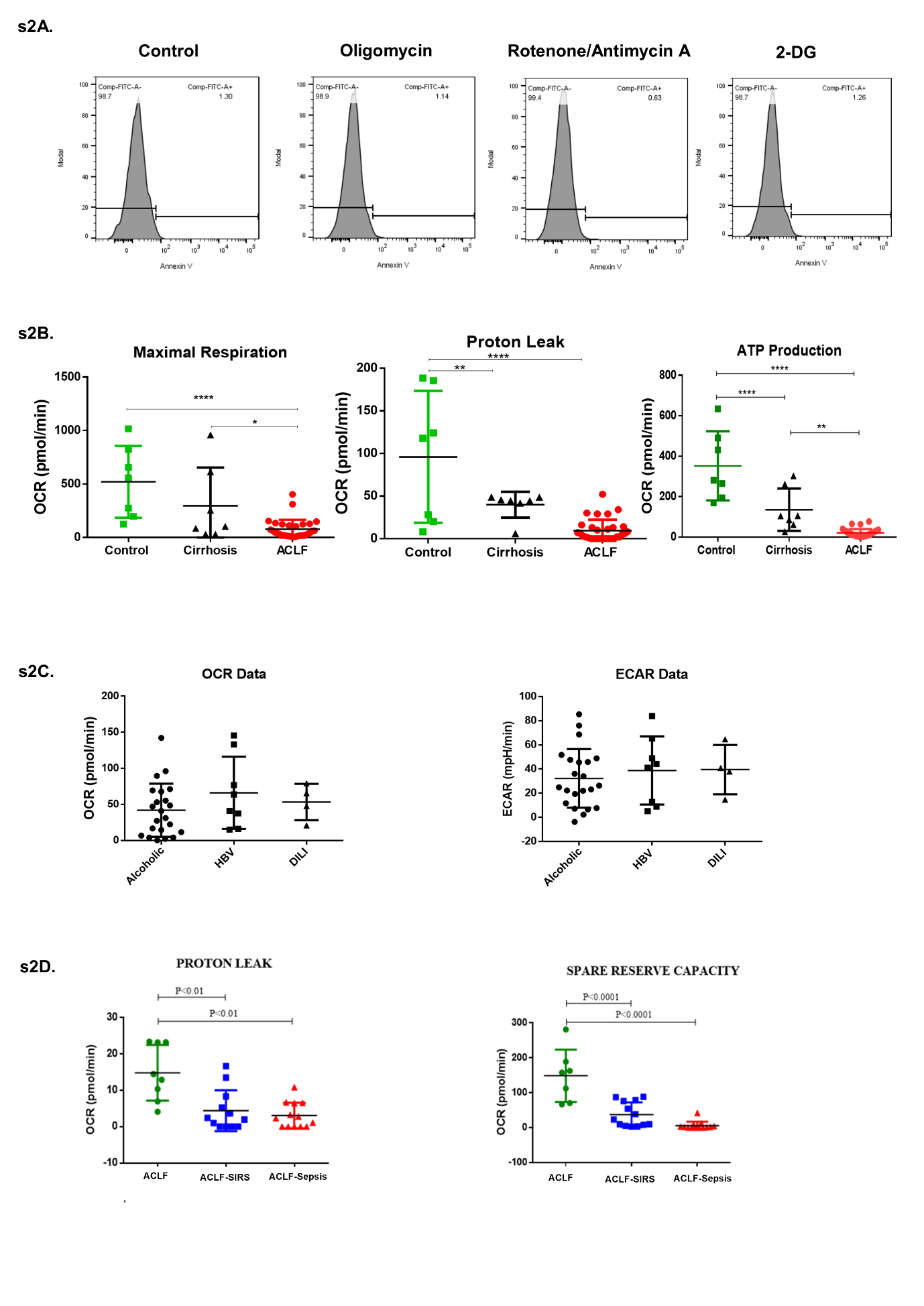
**

**Figure S2: (A)** Effect of Oligomycin, Rotenone/Antimycin A and 2-DG on viability of monocytes assessed by Annexin V staining (B) Dot plots showing changes in mitochondrial parameters (Maximal Respiration, proton leak, ATP production) in healthy vs cirrhosis vs ACLF (C) Dot plot showing changes in mitochondrial basal respiration and baseline glycolysis in different aetiologies of ACLF (ACLF, ACLF-SIRS, ACLF-Sepsis) (D) Dot plot showing changes in proton leak and spare reserve capacity of monocytes from ACLF subsets (ACLF, ACLF-SIRS, ACLF-Sepsis)


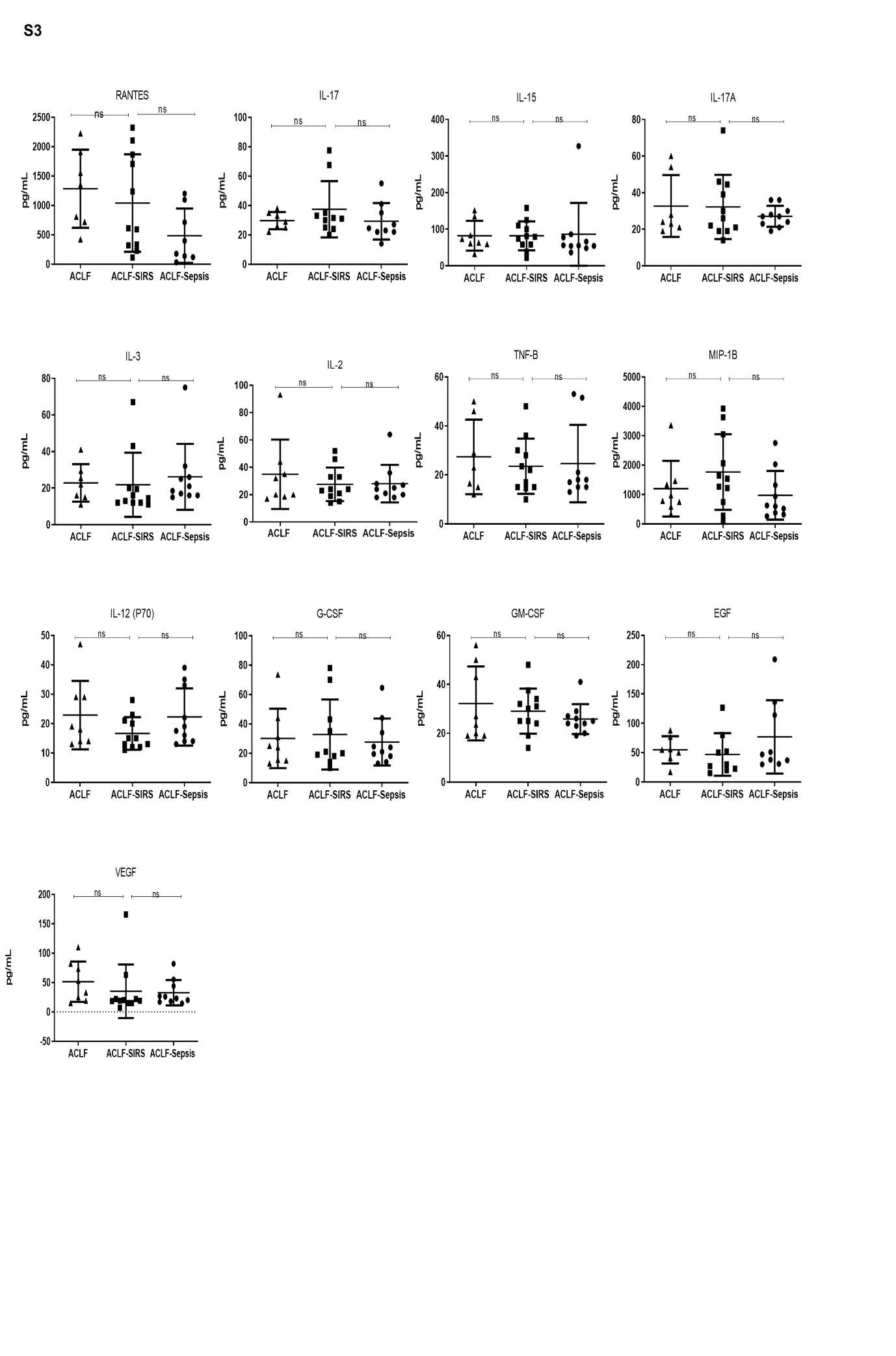
**Figure S3:** Dot plots showing changes in plasma level of cytokines (Rantes, IL-17, IL-15, IL-17A, IL-13, IL-2, TNF-β, MIP-1B, IL-12 (P70), G-CSF, GM-CSF, EGF, VEGF) in ACLF subgroups (No SIRS, SIRS, Sepsis).

**
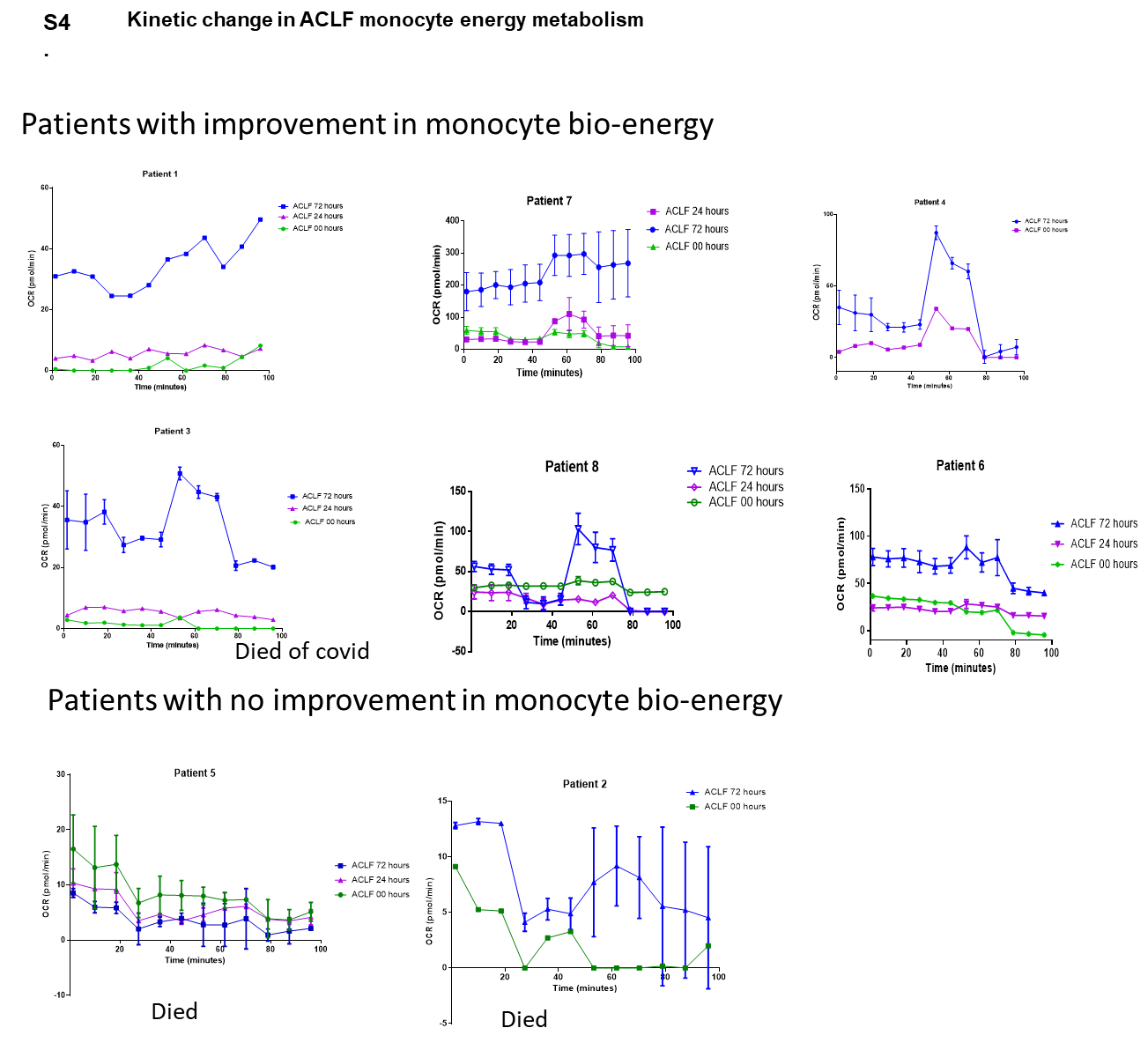
**

**Figure S4:** Graphs showing real time changes in Oxygen Consumption Rate (OCR) with subsequent treatment with oligomycin (Oligo.) FCCP and Rotenone and Actinomycin A (Rot. + Act.) in ACLF (n=8) monocytes at bassline (0h) and 24h and 72h post treatment.


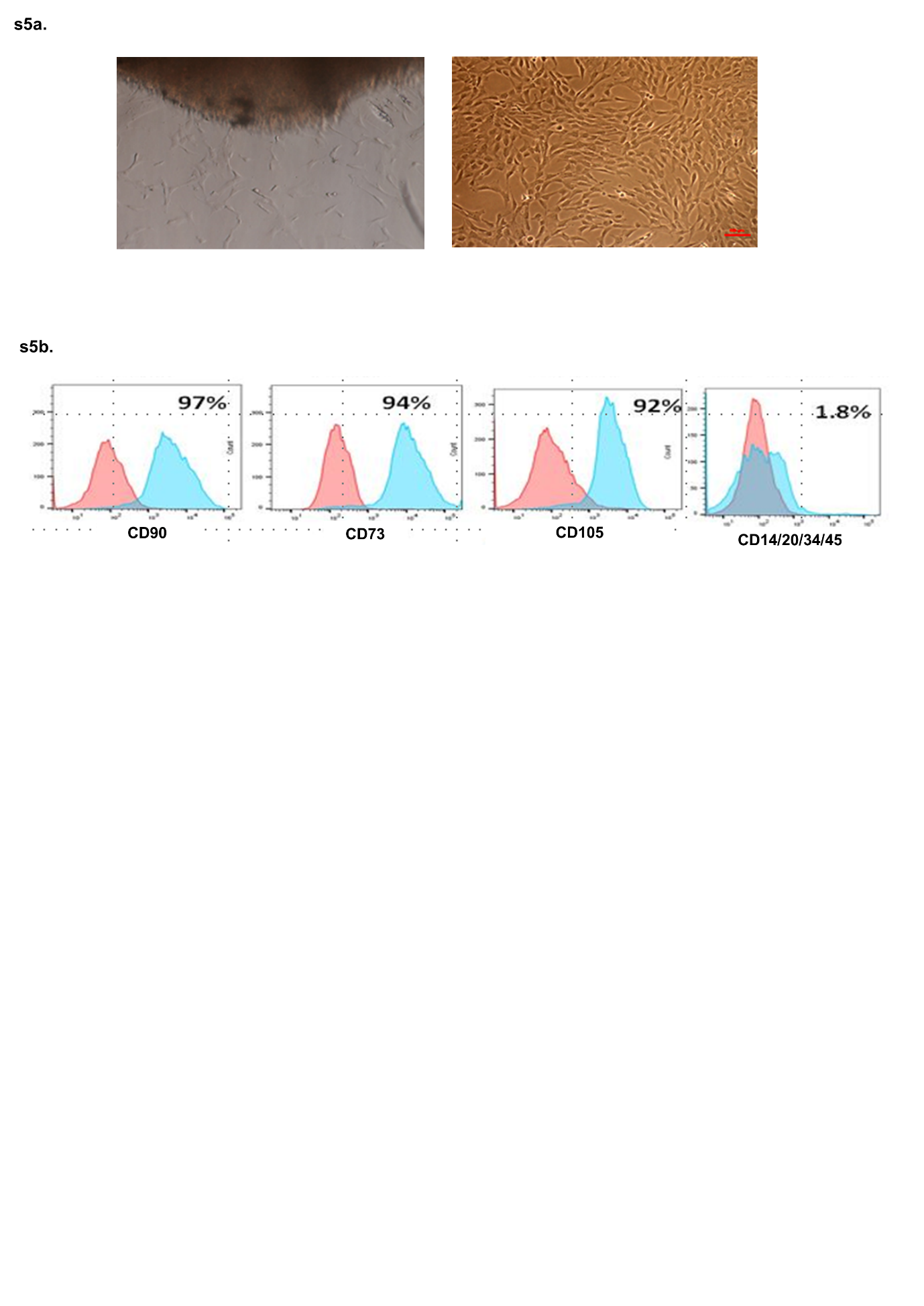


**Figure S5:** (A) Images showing explant culture of Mesenchymal Stem Cells from human Umbilical Cord (left) and spindle shape morphology of passage-3 ucMSC (right) (B) Flowcytometric analysis of ucMSC showing MSC surface marker expression negative markers (CD14, CD20, CD34, CD45) and positive markers (CD90, CD73, and CD105)


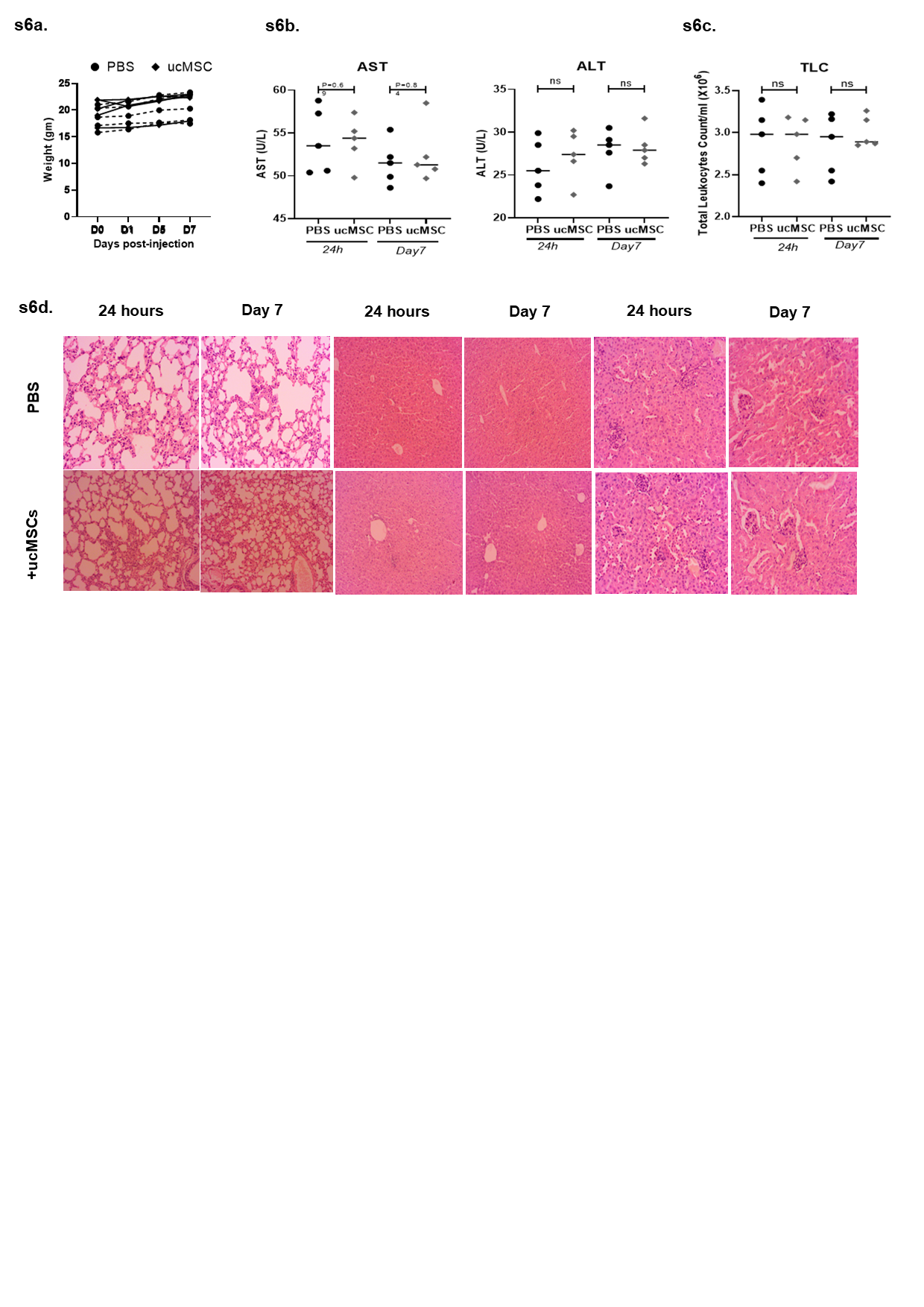


**Figure S6:** (A) Line graph showing change in body weight of healthy C57Bl6 mice (n=5) post ucMSC /PBS infusion (B) dot plot showing change in AST and ALT at 24 h and day 7 post ucMSC /PBS infusion in healthy C57Bl6 mice. (C) dot plot showing change in peripheral blood total leucocyte count (TLC) at 24 h and day 7 post ucMSC /PBS infusion in healthy C57Bl6 mice (D) Representative micrograph showing H&E staining of Lungs, Liver and Kidney Tissue at 24 h and day 7 post ucMSC /PBS infusion in healthy C57Bl6 mice


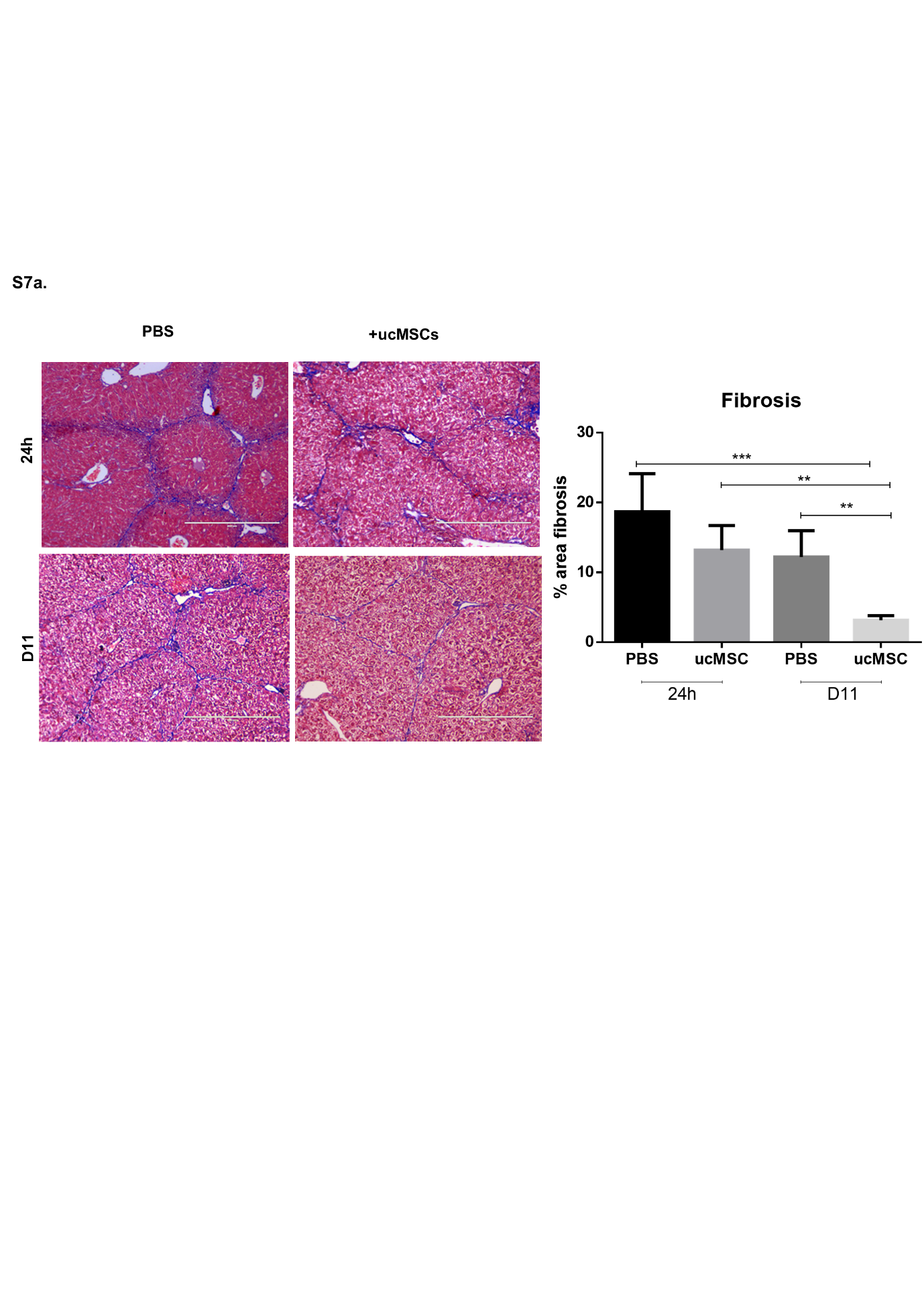


**Figure S7:** Representative images showing level of fibrosis (using Mason’s Trichome staining) at 24 hours and Day 11 in control (PBS treated) and ucMSCs treated ACLF animals. Bar-graphs showing the percent area of fibrosis in control and ucMSCs treated ACLF animal model.
